# A *Legionella* Effector Disrupts Host Cytoskeletal Structure by Cleaving Actin

**DOI:** 10.1371/journal.ppat.1006186

**Published:** 2017-01-27

**Authors:** Yao Liu, Wenhan Zhu, Yunhao Tan, Ernesto S. Nakayasu, Christopher J. Staiger, Zhao-Qing Luo

**Affiliations:** 1 Purdue Institute for Inflammation, Immunology and Infectious Diseases and Department of Biological Sciences, Purdue University, West Lafayette, IN, United States of America; 2 Biological Sciences Division, Pacific Northwest National Laboratory, Richland, WA, United States of America; Yale University School of Medicine, UNITED STATES

## Abstract

*Legionella pneumophila*, the etiological agent of Legionnaires’ disease, replicates intracellularly in protozoan and human hosts. Successful colonization and replication of this pathogen in host cells requires the Dot/Icm type IVB secretion system, which translocates approximately 300 effector proteins into the host cell to modulate various cellular processes. In this study, we identified RavK as a Dot/Icm substrate that targets the host cytoskeleton and reduces actin filament abundance in mammalian cells upon ectopic expression. RavK harbors an H_95_E_XX_H_99_ motif associated with diverse metalloproteases, which is essential for the inhibition of yeast growth and for the induction of cell rounding in HEK293T cells. We demonstrate that the actin protein itself is the cellular target of RavK and that this effector cleaves actin at a site between residues Thr351 and Phe352. Importantly, RavK-mediated actin cleavage also occurs during *L*. *pneumophila* infection. Cleavage by RavK abolishes the ability of actin to form polymers. Furthermore, an F352A mutation renders actin resistant to RavK-mediated cleavage; expression of the mutant in mammalian cells suppresses the cell rounding phenotype caused by RavK, further establishing that actin is the physiological substrate of RavK. Thus, *L*. *pneumophila* exploits components of the host cytoskeleton by multiple effectors with distinct mechanisms, highlighting the importance of modulating cellular processes governed by the actin cytoskeleton in the intracellular life cycle of this pathogen.

## Author Summary

Actin is a core component of the actin cytoskeleton, which plays a crucial role in diverse cellular processes including cell migration, cytokinesis, endocytosis and vesicle trafficking. Therefore, it is not surprising that many pathogens target actin and/or proteins involved in the regulation of actin activity for their benefit. *Legionella pneumophila*, the etiological agent of Legionnaires’ disease, uses the Dot/Icm type IVB secretion system to transfer effectors into host cells to subvert host cellular processes for its intracellular replication. At least three Dot/Icm substrates, VipA, Ceg14 and LegK2 have been shown to modulate the host actin cytoskeleton. Here, by screening *L*. *pneumophila* Dot/Icm substrates that alter the actin cytoskeleton in mammalian cells, we have identified RavK as an additional effector that specifically disrupts actin organization. RavK harbors a canonical metalloprotease motif, which is essential for the RavK-mediated actin cytoskeleton disruption and cell- rounding phenotypes. We further demonstrate that RavK directly cleaves actin, generating a fragment with a diminished capacity to form actin filaments. Our results reveal a new mechanism for which an intravacuolar bacterium disrupts actin cytoskeleton through the cleavage of the actin molecule, rather than interfering with the endogenous actin regulation pathways or by posttranslational modification of the actin molecule, to benefit its intracellular life cycle.

## Introduction

*Legionella pneumophila* is a ubiquitous Gram-negative bacterium that lives as a parasite of fresh water amoebae in the environment. It is also an important pathogen for humans; inhalation of *L*. *pneumophila*-contaminated aerosols by immune-compromised individuals can lead to a severe form of pneumonia, which is referred to as Legionnaires’ disease [1]. It is believed that protozoans hosts provide the major evolutionary pressure for *L*. *pneumophila* to acquire and maintain virulence factors essential for its intracellular survival and replication in human macrophages [2].

One hallmark of *L*. *pneumophila* infection is the formation of an ER-derived membrane-bounded vacuole known as the Legionella-containing vacuole (LCV), which bypasses the default endocytic pathway that ultimately delivers phagocytosed particles to the lysosome. The biogenesis and development of the LCV strictly requires the Dot/Icm type IV secretion system [3,4], through which approximately 300 protein substrates are translocated into the host cytosol. These proteins, also called effectors, function to modulate a wide spectrum of host cellular pathways, including membrane trafficking, ubiquitination, autophagy, immune responses, and the actin cytoskeleton [5–13]. Despite intensive efforts, only a small proportion (about 10%) of the ~300 Dot/Icm effector proteins have been characterized biochemically [14,15].

The 42-kDa actin protein assembles into filaments within cells to construct a pervasive and dynamic cytoskeleton, which plays a crucial role in diverse cellular processes including cell migration, cytokinesis, endocytosis and vesicle trafficking [16]. Therefore, it is not surprising that many pathogens have evolved effective strategies to target actin and/or proteins involved in the regulation of actin activity. Intracellular bacterial pathogens such as species of *Listeria*, *Shigella*, *Rickettsia* and *Burkholderia* take advantage of distinct host actin polymerization machineries to facilitate their movement within the host cytosol and/or their cell-to-cell spread [17]. *Salmonella enterica* Typhimurium modulates the actin cytoskeleton to gain entry into non-phagocytic cells [18]. *Chlamydia trachomatis* coopts the function of actin filaments and intermediate filaments to stabilize its replicative vacuole in epithelial cells [19]. Apart from these, bacterial proteins directly modifying actin monomers have also been identified. The best-studied modification is ADP-ribosylation of actin by the C2 toxin from *Clostridium botulinum*, which modifies Arg-177 of actin, leading to the inhibition of actin polymerization [20]. In contrast, the *Photorhabdus luminescens* Tc toxin ADP-ribosylates the Thr-148 residue to promote actin polymerization, facilitating the formation of actin aggregates [21]. Bacterial proteins that cleave actin have also been identified; the metalloprotease ECP32 from *Serratia proteamaculans* cleaves actin, and ectopic expression of this protein enables nonpathogenic *E*. *coli* to invade eukaryotic cells [22].

Targeting host actin cytoskeleton by *L*. *pneumophila* virulence factors has emerged as an exciting area of research. At least three *Legionella* Dot/Icm substrates have been shown to modulate distinct cell biological aspects of actin cytoskeleton components. VipA is an actin nucleator, which localizes to actin patches and endosomes during infection and promotes actin polymerization [13]; Ceg14 co-sediments with filamentous actin and inhibits actin polymerization by an unknown mechanism [12]; LegK2 is a kinase that phosphorylates ArpC1b and Arp3, two subunits of the Arp2/3 complex, thus inhibiting actin polymerization on the LCV [11]. Considering the importance of the actin cytoskeleton in cellular processes and extensive functional redundancy among *Legionella* effectors, we hypothesized that more Dot/Icm effectors function to target the actin cytoskeleton. In a screening for Dot/Icm substrates capable of modulating the actin cytoskeleton, we identified RavK as an effector that disrupts the actin cytoskeleton of mammalian cells. We further provide evidence that RavK is a zinc-dependent metalloprotease that specifically cleaves actin and abolishes its polymerization activity. Together with earlier reports on VipA, LegK2 and Ceg14, our results add to a growing body of evidence that *L*. *pneumophila* utilizes multiple proteins to modulate different aspects of the host actin cytoskeleton in its intracellular life cycle.

## Results

### RavK is a *Legionella* effector that disrupts the actin cytoskeleton in mammalian cells

**Fig 1:**
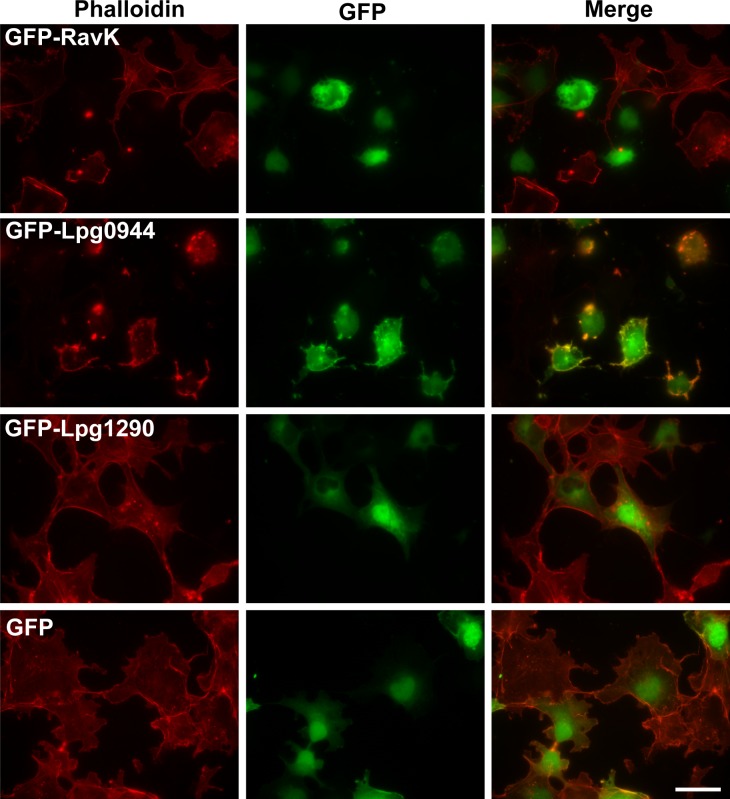
Identification of *L*. *pneumophila* Dot/Icm substrates capable of altering the architecture of the actin cytoskeleton of mammalian cells. COS-1 cells were transfected to express GFP or GFP fusion of RavK, Lpg0944 and Lpg1290 for 24 h and fixed cells were subjected to staining with Texas-red-conjugated phalloidin. Images shown were from one representative experiment and similar results were seen in three independent experiments. Note that RavK severely reduced the phalloidin signals and that GFP-Lpg0944 caused a rearrangement of the actin cytoskeleton with an increase in cortical actin abundance. In contrast, expression of Lpg1290 did not cause any significant change in the actin cytoskeleton. The cells expressing GFP served as a control. Bar, 20 μm.

To identify effectors that target the actin cytoskeleton, we screened a GFP fusion library of Dot/Icm substrates [23] for their ability to alter the morphology of the mammalian actin cytoskeleton. Given the essential role of the actin cytoskeleton in cell viability, disruption of its structure most likely is detrimental; we thus began our screening by examining the effects of Dot/Icm substrates known to be toxic to yeast [12,24–26]. From the first eight candidates screened, we found that ectopic expression of effector RavK (Lpg0969) led to the abolishment of the actin cytoskeleton in COS-1 cells (**Fig 1)**. Interestingly, overexpression of Lpg0944 caused a detectable rearrangement of actin cytoskeleton with more F-actin accumulating on the plasma membrane (**Fig 1)**. In contrast, cells transfected to express the other 6 effectors showed only very minor or undetectable changes in the structure of the actin cytoskeleton compared with those expressing GFP (**Fig 1, S1 Fig**). The strong and clear phenotype associated with RavK prompted us to further investigate its mechanism of action.

### RavK is a substrate of the Dot/Icm transporter whose expression is induced at the exponential phase

RavK was originally identified in a screening for *L*. *pneumophila* Dot/Icm substrates by its ability to restore the translocation of the transfer-deficient mutant SidCΔC100, and therefore was designated as RavK (*r*egion *a*llowing *v*acuole co-localization K) [27]. Dot/Icm-dependent translocation of RavK was independently demonstrated using the CCF4/β-lactamase reporter assay [5].

**Fig 2:**
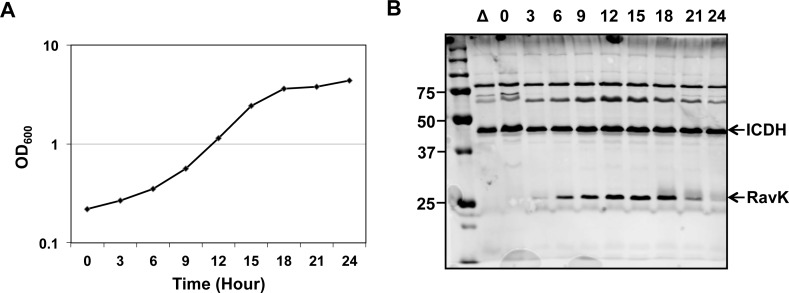
Expression of RavK is induced in the exponential phase of bacterial growth. **A**. The growth of *L*. *pneumophila* in AYE broth. Cultures grown to stationary phase were diluted 1:20 into fresh medium and the growth of bacteria was monitored by measuring OD_600_ at the indicated time points. **B**. RavK protein level peaked at exponential growth phase. Lysates were prepared from equal amount of cells withdrawn at the indicated time points and were resolved by SDS/PAGE, and the levels of RavK were examined by immunoblotting with a RavK-specific antibody. The metabolic protein isocitrate dehydrogenase (ICDH) was probed as a loading control.

Probably due to the need for effectors that effectively thwart the host defense in the initial phase of infection, the expression of many Dot/Icm substrates is induced during the post-exponential phase, when *L*. *pneumophila* concomitantly enters the transmissive phase and becomes primed for a new round of infection [24]. We therefore examined the level of RavK at different time points throughout the growth cycle of *L*. *pneumophila* in broth. Interestingly, the expression of RavK was highly induced in exponentially growing bacteria (6–18 h) (OD_600_ between 0.4 and 3.0); the protein was barely detectable in the lag (0–6 h) or the post-exponential phase (18–24 h) (**Fig 2**), which indicates that RavK likely plays a role in the replicative phase during *L*. *pneumophila* infection.

### An HE_XX_H motif is essential for the toxicity of RavK and its disruption of the host cytoskeleton

**Fig 3:**
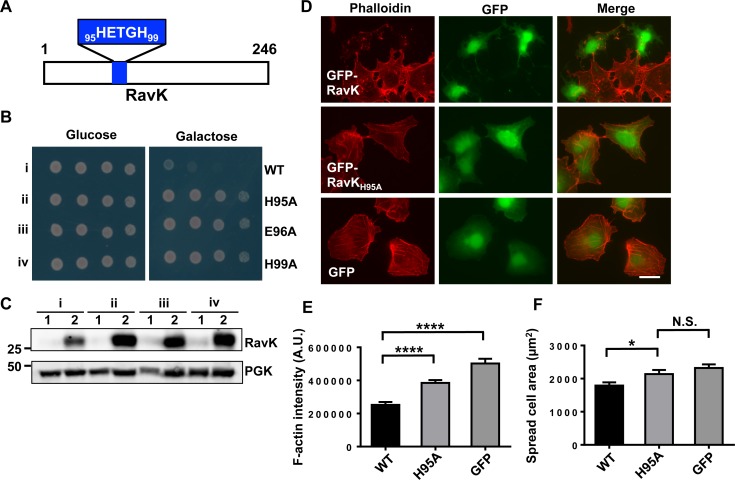
Expression of RavK causes cytotoxicity in both yeast and mammalian cells and reduces the F-actin content in mammalian cells. **A**. A schematic diagram of RavK. The blue box highlighted the position and the sequence of the predicted HE_XX_H motif. **B**. Expression of RavK induces yeast growth arrest in an H_95_E_XX_H_99_-dependent manner. Yeast strains expressing RavK or the indicated mutants under the control of the galactose-inducible promoter were serial-diluted and spotted onto plates containing glucose or galactose, respectively. Plates were incubated at 30°C for 48 h before image acquisition. **C**. Expression of RavK and the indicated mutants in yeast. Yeast strains grown in glucose medium to saturation were washed with water 5 times and split equally to 2 halves. One half was frozen immediately (sample 1), the other half was induced in galactose medium for 8 h (sample 2). Total proteins of all samples were resolved by SDS/PAGE and probed by immunoblotting with a RavK-specific antibody. The 3-phosphoglycerate kinase (PGK) was used as a loading control. **D**. RavK reduces F-actin content in COS-1 cells. COS-1 cells transfected by the indicated plasmids for 24 h were fixed and subjected to Texas-red-conjugated phalloidin staining. Images from one representative were shown and similar results were obtained in at least three experiments. Bar, 20 μm. E. Integrated pixel density of phalloidin staining in cells expressing indicated proteins plotted as average F-actin intensity per cell. N>60 per condition; error bars represent standard error of the mean (SEM); A.U., arbitrary units; ****, *p*<0.0001. F. The spread cell area of cells expressing indicated proteins plotted as average area per cell. N>60 per condition; N.S., not significant; *, *p*<0.05.

To understand the mechanism of action of RavK, we first performed sequence analysis of the protein to search for the presence of motifs suggestive of known biochemical activity. We manually scanned the sequence of RavK against the “PROSITE collection of motifs” [28], and found that RavK harbors an H_95_E_XX_H_99_ motif present in diverse metalloproteases [29] (**Fig 3A**). To determine the role of this motif in the activity of RavK, we introduced mutations in H_95_, E_96_ and H_99,_ respectively. Next we assessed the effects of these mutations on the activity of RavK by examining their toxicity to yeast; while not affecting the stability of RavK, each of these mutations completely abolished the toxicity to yeast (**Fig 3B and 3C**). To examine whether the H_95_E_XX_H_99_ motif is required for the disruption of actin cytoskeleton, we expressed GFP-RavK, GFP-RavK_H95A_ or GFP in COS-1 cells and labeled the actin cytoskeleton with Texas-red-conjugated phalloidin. Relative F-actin levels in transfected cells were analyzed by calculating the integrated pixel density of phalloidin fluorescence of outlined individual cell. Our results indicate that the total F-actin levels in GFP-RavK-expressing cells were significantly lower than those in cells expressing GFP or GFP-RavK_H95A_ (**Fig 3D and 3E**). We also found that cells expressing GFP-RavK were significantly smaller than that those expressing GFP or GFP-RavK_H95A_ (**Fig 3F**), indicating that ectopic expression of RavK caused shrinkage in COS-1 cells.

In comparison to the RavK-mediated morphological alterations in COS-1 cells, ectopic expression of GFP-RavK caused a clear cell-rounding phenotype in HEK293T cells. Consistently, the observed phenotype in HEK293T cells also depends on the H_95_E_XX_H_99_ motif (**S2A Fig**). The different responses to RavK by COS-1 and HEK293T cells may be due to the expression level, variations in the cellular level of the protein targeted by RavK, or a combination of both. Nevertheless, these results suggest that RavK is a metalloprotease that potentially target components of the host cytoskeleton.

### Expression of RavK reduces the total actin level in mammalian cells

**Fig 4:**
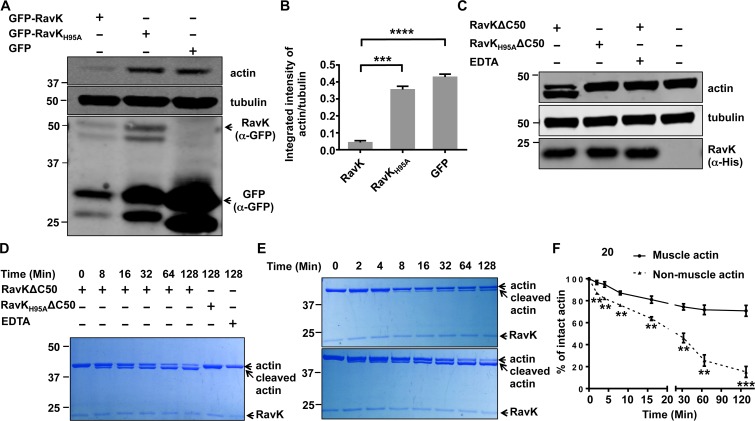
The cleavage of actin by RavK. **A**. Expression of RavK reduced the level of actin in COS-1 cells. Indicated proteins were expressed in COS-1 cells for 24 hours and cleared cell lysates were subjected to SDS/PAGE and immunoblotting with GFP-specific and actin-specific antibodies, respectively. α-tubulin was probed as a loading control. **B**. The intensity of the bands corresponding to actin and tubulin was measured with ImageJ and the intensity ratio between actin and tubulin revealed the relative actin level in cells of the relevant samples. All results are from three independent experiments. Error bars represent standard error of the mean (SEM). ***, *p*<0.001, ****, *p*<0.0001. **C**. Recombinant RavKΔC50 cleaved actin in lysates of COS-1 cells. Indicated proteins were added to COS-1 cell lysates and the reactions were allowed to proceed for 1 h at 22°C, EDTA was added to the indicated samples. Samples were separated by SDS/PAGE and detected by immunoblotting with an actin-specific antibody. α-tubulin was probed as a loading control. **D**. Recombinant RavKΔC50 cleaved actin *in vitro* in an HE_XX_H motif-dependent manner. 10-μg human non-muscle actin was incubated with 1-μg RavKΔC50 or RavK_H95A_ΔC50 for the indicated time and the mixtures separated by SDS/PAGE were stained with Coomassie brilliant blue. EDTA was added into the samples at the beginning when indicated. **E**. The cleavage of human non-muscle actin and rabbit muscle actin by RavKΔC50. The *in vitro* cleavage was performed similarly as described in D. Upper panel: Muscle actin; Lower panel: Non-muscle actin. F. RavKΔC50 cleaves human non-muscle actin more efficiently than rabbit muscle actin. The percentage of intact actin/total actin was calculated with ImageJ. All results were from three independent experiments. *, *p*<0.05, ***, *p*<0.001.

Actin exists in cells as both free monomer, called G-actin (globular actin), and as polymeric microfilaments, called F-actin (filamentous actin) [30]. The RavK-induced reduction of the phalloidin-stainable F-actin in COS-1 cells can be accounted for by at least two possibilities. First, RavK directly reduces the total pool of actin within the cells by mechanisms such as proteolytic cleavage. Second, RavK somehow tilts the balance toward G-actin and reduces the pool of F-actin. To distinguish between these two possibilities, we compared the total actin level between cells expressing RavK and the RavK_H95A_ mutant and found that cells expressing wild-type RavK contained much lower levels of total actin than that of cells expressing RavK_H95A_ or GFP (**Fig 4A and 4B**), indicating that RavK reduces the abundance of total actin in COS-1 cells. Similarly, RavK expression also reduced total actin level in HEK293T cells (**S3A Fig**).

### RavK is a protease that cleaves actin

The reduction of cellular actin levels by RavK can be caused by directly degrading the protein or by initiating a signaling cascade that leads to lower cellular actin levels. A direct approach to distinguish between these two models is by incubating recombinant RavK with total lysates of mammalian cells and examining the levels of actin. To obtain active RavK protein for such biochemical assays, we made numerous attempts to express epitope-tagged RavK for affinity purification from *E*. *coli*, none of the used tags such as His_6_, His_6_-Sumo, and GST allowed us to obtain soluble full-length RavK (**S4A Fig**). We therefore initiated a screening to identify truncated alleles of RavK that would potentially be soluble and functional for biochemical studies. A series of RavK deletion mutants were constructed by removing residues from its C-terminal end (the H_95_E_XX_H_99_ motif localizes toward its N-terminal portion). Whereas deletion of 50 residues from the C-terminal end led to a mutant that retained the toxicity to yeast, a mutant lacking 100 amino acids from the same end abolished its toxicity (**S4B and S4C Fig**). Consistent with its toxicity to yeast, RavKΔC50 still caused cell rounding in HEK293T cells (**S4D and S4E Fig**). Notably, the ΔC50 deletion greatly increased the solubility of RavK, which allowed us to obtain sufficient recombinant protein for biochemical experiments (**S4A Fig**).

To determine the activity of the recombinant RavKΔC50, we incubated lysates of COS-1 cells with His_6_-RavKΔC50 or His_6_-RavKΔC50_H95A_ at 22°C for 1 h. Wild type RavKΔC50 but not the H95A mutant caused a reduction of full-length actin and produced an actin fragment clearly smaller than the original protein in the cell lysates. Furthermore, the reduction of actin can be inhibited by the metal ion chelator EDTA (**Fig 4C**). Similar results were observed with lysates of HEK293T cells (**S3C Fig**). Thus, RavK is a metalloprotease, which is able to cleave actin in lysates of mammalian cells in an H_95_ExxH_99_ motif-dependent manner.

We next tested whether any host factor is required for the cleavage of actin by RavK by mixing human non-muscle actin (85% β-actin and 15% γ-actin) with His_6_-RavKΔC50 or His_6_-RavKΔC50_H95A_ for various time durations. Incubation with His_6_-RavKΔC50 but not with the H95A mutant produced a smaller actin fragment (**Fig 4D**) and the size difference between these two fragments is similar to that observed in experiments using total cell lysates. Consistent with earlier observations, the activity of RavK is sensitive to EDTA. Thus, RavK is a metalloprotease that cleaves actin without the requirement of any other host proteins.

Among the three major groups of actin (α, β, γ) identified in vertebrates, the α-actin is the major constituent of the contractile apparatus in muscle cells, whereas the β and γ actin coexist in most of non-muscle cells as a component of cytoskeleton [31]. We thus tested whether RavK has a preference toward specific actin isoforms. Since the protein sequence of commercially available rabbit skeletal muscle actin is identical to that of human skeletal muscle actin, we used rabbit muscle actin in this assay for comparison to human non-muscle actin. The same amount of rabbit muscle actin and human non-muscle actin was treated with equal amount of His_6_-RavKΔC50. As early as 2 min post treatment, significantly more cleaved product was detected in reactions with non-muscle actin than those with muscle actin. When the reaction was allowed to proceed for 128 min, more than 80% of non-muscle actin was cleaved, whereas only approximately 25% of muscle actin that was cleaved in this experimental duration (**Fig 4E and 4F)**. Thus, RavK cleaves the non-muscle actin more efficiently than the muscle actin.

As RavK harbors an H_95_E_XX_H_99_ motif that is common for zinc binding, we further tested whether zinc is required for the activity of RavK. Seven different metal ions including zinc were tested for their ability to restore the activity of metal ion-free RavK. Indeed, Zn^2+^ was able to restore the activity of RavK (**S5 Fig**). Notably, whereas such divalent ions as Co^2+^, Ni^2+^, Cu^2+^, Ca^2+^ or Mg^2+^ cannot detectably restore the activity of RavK, Mn^2+^ was able to restore the activity of RavK at levels comparable to those of Zn^2+^, probably due to their similarity in chemical properties [32].

### RavK-dependent cleavage of actin during *L*. *pneumophila* infection

**Fig 5:**
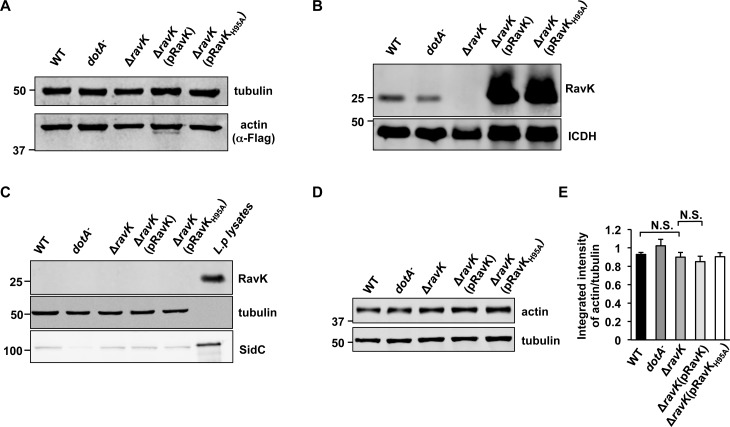
RavK-mediated cleavage of actin occurs during *L*. *pneumophila* infection. **A**. Actin is cleaved by translocated RavK during bacterial infection. Cells expressing Flag-tagged actin were infected with relevant *L*. *pneumophila* strains for 2 h and cleared cell lysates was probed by immunoblotting with a Flag specific antibody. Tubulin was probed as a loading control. Similar results were obtained from five independent experiments and a representative blot was shown. **B**. The detection of RavK expression in *L*. *pneumophila*. The lysates of WT and the *dotA*^*-*^ mutant were from exponentially grown bacteria whereas those of other strains were from bacteria grown at the post-exponential phase; the metabolic enzyme isocitrate dehydrogenase (ICDH) was probed as a loading control. **C**. RavK cannot be detected in saponin-soluble fractions of infected cells with RavK-specific antibody. SidC, a known Dot/Icm substrate was probed as a positive control for translocation and tubulin was probed as a loading control. **D**. Cleavage of endogenous actin. Lysates of cells similarly infected as described in C were probed for actin with tubulin as a loading control (left panel). **E**. The ratio of intensity between the bands representing actin and tubulin was quantified from three independent experiments. Note that a reduction in actin was not observed even in infections using the RavK overexpressing strain. N.S., not significant.

To investigate whether the RavK-mediated cleavage of actin occurs during bacterial infection, we infected mammalian cells expressing 4xFlag-tagged actin with wild-type *L*. *pneumophila* or its derivatives. Cleaved actin was only detected in samples infected with the *ΔravK* strain expressing RavK from a multi-copy plasmid but not in samples infected by the *ΔravK* strain or *ΔravK* overexpressing RavK_H95A_, indicating that actin is cleaved during *L*. *pneumophila* infection in a RavK-dependent and more specifically metalloprotease motif-dependent manner (**Fig 5A**). The cleaved form of actin was not observed in samples infected by wild-type *L*. *pneumophila*, which may be attributed to less translocated RavK in host cytosol compared to samples infected by *ΔravK* overexpressing RavK. Considering the expression level of RavK is much higher in *ΔravK* overexpressing RavK than in wild-type *L*. *pneumophila* (**Fig 5B**), it is almost certain that more RavK was translocated to host cells by the overexpressing strain, which caused detectable cleavage of Flag-actin. Yet, in both cases the amount of translocated RavK was below the detection capacity of immunoblotting with our RavK-specific antibody (**Fig 5C**). Despite multiple attempts using different infection conditions such as variations in multiplicity of infection (MOI), infection time and host cells, we were unable to detect the cleavage of endogenous actin even in infections using the strain overexpressing RavK (**Fig 5D**). The inability to detect the reduction of actin or the cleaved product may attribute to low stability of the cleaved product in cells, the quality of the antibodies used for detection or the potential compensatory effects from the hosts, or a combination of these factors.

### Cleavage of actin by RavK occurs at a site between T351 and F352

**Fig 6:**
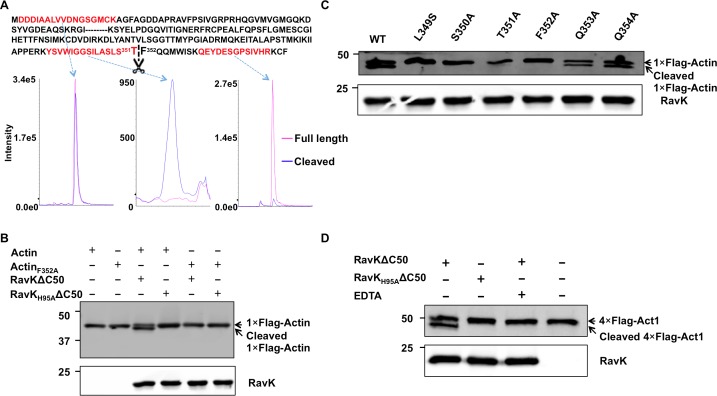
RavK cleaves β-actin by recognizing a site between T351 and F352. **A**. RavK cleaved β-actin at a site between T351 and F352. A portion of actin amino acid sequence containing the relevant peptides was shown. A comparison of the abundance of the semi-tryptic end peptide -YSVWIGGSILASLST- in two bands suggested that the cleavage site lies between T351 and F352. The abundance of two fragments located in the N-terminal and the C-terminal portion of actin, respectively, was compared to validate the results. **B**. The β-actin F352A mutant was resistant to RavK cleavage. HEK293T cells were transfected to express Flag tagged β-actin WT or its F352A mutant. 24 h after transfection, cell lysates were subjected to immunoprecipitation using M2 beads for 3 h. Proteins eluted with 3×Flag peptides were treated by either RavKΔC50 or RavK_H95A_ΔC50 for 2 h and the samples were separated by SDS-PAGE and detected by immunoblotting with Flag-specific antibody. **C**. The Q353A and Q354A mutants of β-actin were still sensitive to RavK. Residues at the indicated positions were mutated and the mutant proteins were individually expressed in HEK293T cells, Flag-tagged proteins obtained as described in (B) were treated with RavKΔ50WT and were subjected to immunoblotting. **D**. The cleavage of yeast actin by RavK. Flag-tagged Act1 expressed in yeast obtained from cell lysates by immunoprecipitation was subjected to RavK cleavage. Samples were separated by SDS-PAGE and detected by immunoblotting with Flag-specific antibody and His_6_-specific antibody, respectively.

To determine the cleavage site of actin by RavK, we incubated non-muscle actin with His_6_-RavKΔC50 at 22°C for 1 h. Samples resolved by SDS-PAGE were detected by Coomassie brilliant blue staining. Protein bands corresponding to both the uncleaved, full-length and the cleaved products were excised, digested with trypsin and sequenced by mass spectrometry (**S6A Fig**). Analysis of the detected tryptic fragments revealed that the semi-tryptic peptide -Y_337_SVWIGGSILASLST_351_- was present in the cleaved protein but not in the full length protein, suggesting that the cleavage site lies between Thr351 and Phe352 (**Fig 6A**). Consistent with this notion, the abundance of the N-terminal peptide -D_2_DDIAALVVDNGSGMCK_18_- was similar between these two proteins, whereas the abundance of the C-terminal peptide -Q_360_EYDESGPSIVHR_372_- was significantly higher in the full-length protein than in the cleaved product (**Fig 6A**), further suggesting that the cleavage site identified by this method is reliable. We confirmed the identified cleavage site by mutating Phe352 into an Ala in β-actin. A Flag-tagged β-actin_F352A_ gene was expressed in HEK293T cells by transfection. Immunoprecipitated Flag-β-actin_F352A_ eluted with the Flag peptide was incubated with His_6_-RavKΔC50 and the cleavage product was detected by immunoblotting with the Flag-specific antibody. RavK treatment of similarly purified wild type Flag-β-actin yielded two protein bands with a molecular weight difference resemblying that observed in experiments with purified actin (**Fig 6B**). In contrast, only one single protein corresponding to the size of uncleaved actin was detected in samples expressing the actin_F352A_ mutant (**Fig 6B**), establishing that Phe352 is important for RavK-mediated cleavage. Residues around the cleavage site often provide the strucutral context important for recognition by proteases [33]; we therefore constructed a series of mutants with substitution mutations in sites adjacent to Phe352 and examined their sensitivity to RavK. Our results indicate that Leu349, Ser350, Thr351 are indispensible for RavK-mediated cleavage, whereas Gln353 and Gln354 are not essential (**Fig 6C**).

With the exception of Ser350, which is replaced by a Thr residue in yeast actin, all the other residues tested in our mutational analysis are conserved among all human actin isoforms and yeast actin (Act1) (**S6B Fig**). Thus, it is likely that the yeast toxicity of RavK is due to its cleavage of yeast actin. We tested this hypothesis by incubating Flag-Act1 with RavKΔC50. Incubation of wild-type RavK but not the RavK_H95A_ resulted in the production of a smaller Act1 fragment, and the fragment was absent in the reaction receiving EDTA (**Fig 6D**), indicating that RavK cleaves Act1 in a metalloprotease activity-dependent manner. Thus, the protease activity against actin attributes to the cytotoxicity of RavK in yeast.

### The actin_F352A_ mutant suppresses the RavK-induced cell rounding

**Fig 7:**
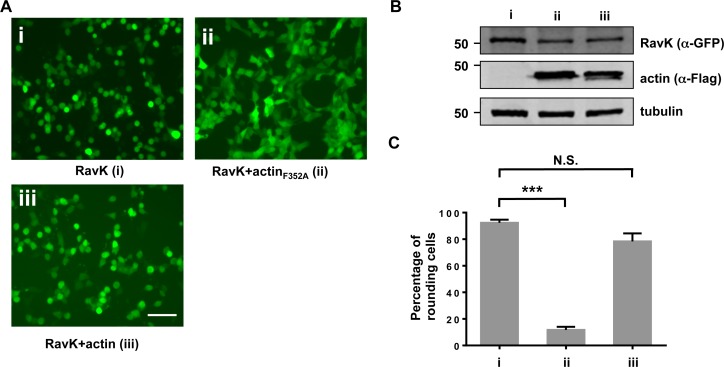
Actin_F352A_ suppresses the cell rounding phenotype caused by RavK. **A**. Actin_F352A_ but not wild-type actin suppressed the cell rounding phenotype caused by RavK. HEK293T cells were transfected with (i) pE*gfp*-*ravK* alone, (ii) pE*gfp*-*ravK* together with pCMV-Flag-*actin* or (iii) pE*gfp*-*ravK* together with pCMV-Flag-*actin*_*F352A*_ for 24 h, and were observed under a fluorescence microscope. Bar, 50 μm. **B**. Expression of GFP-RavK, Flag-actin or Flag-actin_F352A_. Cells were transfected by indicated plasmids for 24 h, and the cleared lysates from transfected samples were resolved by SDS/PAGE, and subjected to immunoblotting with antibodies specific to GFP and the Flag tag, respectively. α-tubulin was used as a loading control. **C**. Quantification of the percentage of green cells exhibiting the cell rounding phenotype. Experiments were performed in triplicate and at least 200 cells were examined in each sample. Error bars indicate standard error of the mean (SEM); N.S., not significant; ****, *p*<0.0001. Similar results were obtained in three independent experiments.

Actin is one of the most abundant proteins in eukaryotic cells, it is possible that the cleavage by RavK we observed is due to non-specific activity. To test whether actin is a *bona fide* target of RavK, we set out to examine whether overexpressing a cleavage-resistant actin variant in HEK293T cells could rescue the cell rouding phenotype mediated by RavK. Overexpression of the actin_F352A_ mutant did not cause any disernable effects in mammalian cells, suggesting that this mutation did not overtly affect the function of actin. If actin is the true substrate of RavK, cells overexpressing actin_F352A_ should become resistant to damage caused by the protease. Overexpression of wild-type actin did not reduce the percentage of rounded cells induced by RavK, which was similar to samples receiving only the construct for RavK (**Fig 7**). In contrast, overexpression of actin_F352A_ in HEK293T cells almost completely abrogated the cell rounding phenotype, although these cells expressed RavK at levels similar to other samples (**Fig 7**). The ability of actin_F352A_ to effectively suppress the RavK-induced phenotypes further establishes that actin is a *bona fide* cellular target of RavK.

### Actin cleaved by RavK is defective in polymerization

**Fig 8:**
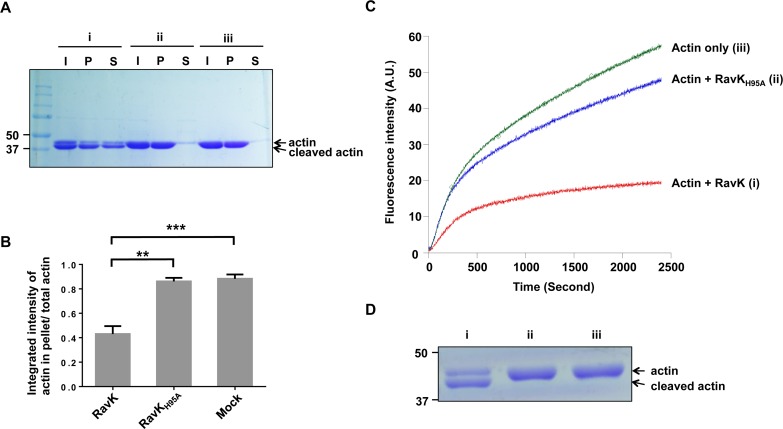
Cleavage by RavK inhibits actin polymerization. **A**. Cleavage by RavK inhibited actin polymerization in actin sedimentation assay. 30-μg non-muscle actin (Cytoskeleton, 99% purity) was incubated with 5-μg wild-type RavKΔC50 or the catalytically dead mutant RavK_H95A_ΔC50 at 23°C for 2 h. The mixtures were precleared by centrifugation at 100,000*g* for 30 min and further used in actin sedimentation assays. Actin polymerization was allowed to proceed for 60 min, followed by ultracentrifugation at 100,000*g* for 40 min. The resulting supernatants and pellets were analyzed by SDS-PAGE followed by Coomassie brilliant blue staining. Groups: i, actin treated with RavKΔC50; ii, actin treated with RavK_H95A_ΔC50; iii, actin without treatment. Lanes: I, input; P, pellet; S, supernatant. **B**. Quantification of the percentage of polymerized actin versus total actin. The band intensity was quantified with ImageJ. All results are obtained from three independent experiments. Error bars represent standard error of the mean (SEM). **, *p*<0.01. **C**. Cleavage by RavK inhibited actin polymerization in a kinetic assembly assay. Non-muscle actin treated by RavKΔC50, RavK_H95A_ΔC50 or a control reaction with RavK was precleared by centrifugation at 100,000*g* for 30 min. 2.7-μM differently-treated actin and 0.3 μM pyrene-labeled actin were mixed and the fluorescence intensity of pyrene (arbitrary units, a.u.) was plotted versus time after the addition of a polymerization buffer to initiate the polymerization. **D**. Actin used in pyrene-labeled actin polymerization assay. Actin samples receiving indicated treatment were resolved by SDS-PAGE and detected by Coomassie brilliant blue staining.

Actin exists as both G-actin and F-actin in the cell and the transition between these two forms in response to cellular needs is precisely regulated [30]. Given the importance of actin polymerization in its function, we tested whether the RavK-cleaved actin retains the ability to form actin filaments. The formation of filaments by G-actin can occur spontaneously under certain conditions, which can be measured by sedimentation after high-speed centrifugation [34]. We therefore determined the polymerization activity of actin after RavK-mediated cleavage. As complete cleavage of non-muscle actin by RavK cannot be achieved even after extended incubation, a mixture consisting of cleaved and uncleaved actin was used in this and following assays. Non-muscle actin that had been incubated with RavKΔC50 or RavK_H95A_ΔC50 at 22°C for 2 h was induced to polymerize for 60 min. The formation of actin filaments was determined by its presence in pellets after ultracentrifugation. Similar to mock-treated actin, in reactions containing actin that had been incubated with RavK_H95A_ΔC50, the majority of actin was in the pellets, indicative of robust polymerization (**Fig 8A and 8B**). In contrast, in reactions containing RavKΔC50, only approximately 40% of the actin was present in the pellet, indicating the cleaved product is defective in polymerization.

To further validate our findings based on the high-speed centrifugation assay, we examined the ability of actin_1-352_ to form actin filaments by a pyrene-labeled actin nucleation assay. Two-hour treatment with RavK_H95A_ΔC50 only slightly decreased the polymerization property of non-muscle actin compared with the control samples receiving no additional protein. On the other hand, incubation with RavKΔC50 for the same time duration drastically reduced the ability of actin to form polymers for non-muscle actin **(Fig 8C and 8D)**, further confirming RavK-cleaved actin is defective in forming actin filaments.

### RavK is not required for proficient intracellular growth of *L*. *pneumophila* in macrophages and a protozoan host

RavK is present in 23 out of 41 sequenced *Legionella* species, and is one of the 74 effectors which are shared by more than 20 different *Legionella* species [35]. Such a high prevalence suggests an important role of this protein in the interactions of the bacteria with their hosts. To study the role of RavK during *L*. *pneumophila* infection, we constructed an in-frame deletion mutant of this gene and investigated the intracellular replication of this mutant in primary mouse macrophages and the protozoan host *Dictyostelium discoideum*. Similar to many Dot/Icm substrates, the results show that the absence of RavK does not affect the uptake by host cells or the intracellular replication capacity of *L*. *pneumophila* in mouse macrophages or *D. discoideum* (**S7A and S7B Fig**). It has been reported that two other *Legionella* effectors Ceg14 and VipA also target host actin cytoskeleton. Ceg14 co-sediments with filamentous actin and inhibits actin polymerization [12], whereas VipA is an actin nucleator, promoting actin polymerization [13]. Since both Ceg14 and RavK negatively affect the actin polymerization, we investigated whether the absence of both of them would cause any intracellular growth defects. The intracellular growth of *ceg14*/*ravK* double knockout strain was determined and the results showed that it still grows as proficiently as the wild-type strain in either mouse macrophages or *D. discoideum* (**S7A and S7B Fig**). Recently, another *Legionella* effector LegK2 was shown to inhibit actin polymerization by phosphorylating the Arp2/3 complex [11]. We therefore made a *ceg14*/*ravK/legk2* triple knockout mutant to examine the potential functional redundancy of these three proteins. The *ceg14*/*ravK/legK2* triple mutant manifests ~10 fold growth defect compared to wild-type strain in *D*. *discoideum*. However, this defect is comparable to that of the *legK2* mutant (**S7C Fig**), indicating the absence of RavK and Ceg14 does not confer any further growth defects to the *legK2* mutant. Furthermore, the intracellular growth defect of the triple mutant can be complemented by *legK2* but not *ravK* or *ceg14* (**S7D Fig**).

## Discussion

Actin cytoskeleton is a common target exploited by many bacterial pathogens, both intra- and extracellular. Generally, bacterial effectors identified to date modulate host actin cytoskeleton by two different mechanisms of action. First, many bacterial effectors interfere with endogenous actin regulation pathways. Examples in the group include effectors that target the small GTPases Rho, Rac and Cdc42, master regulators of the actin cytoskeleton, by either distinct post-translational modifications [36–40], or the regulation of their GTP binding status [41–43]. The second mechanism of action is to directly modify the actin molecule by means of posttranslational modifications such as ADP-ribosylation, or by crosslinking or proteolysis. ADP-ribosylation of actin leads to either promotion or inhibition of actin polymerization depending on the residues being modified. ADP-ribosylation of Arg-177 by the C2 toxin from *Clostridium botulinum* inhibits actin polymerization [20], in contrast, the same modification of Thr-148 by the Tc toxin from *Photorhabdus luminescens* promotes actin polymerization [21]. Actin cross-linking proteins secreted by *Vibrio* and *Aeromonas* species induce the production of actin oligomers that strongly inhibit Formin-mediated actin polymerization [44]. Proteolysis of actin by bacterial proteins has also been documented; the metalloprotease ECP32 from *Serratia grimesii* cleaves actin, and ectopic expression of this protein enables nonpathogenic *E*. *coli* to invade eukaryotic cells [22]. In this study, we have shown that the *Legionella* Dot/Icm substrate RavK is a zinc-dependent metalloprotease that specifically cleaves actin to disrupt the actin cytoskeleton of host cells. Unlike ECP32 from *Serratia*, RavK does not affect the uptake of bacteria (**S7A and S7B Fig)**. Instead, RavK is likely to play a role in the replicative phase of *L*. *pneumophila* during infection, which is supported by the high level expression of *ravK* at the exponential growth phase in bacteriological medium (**Fig 2A and 2B**).

The activity of RavK toward actin generates products that can be further degraded in the cell, thereby causing the reduction of total actin levels (**Fig 4A–4C**), which may explain our inability to detect cleaved actin during *L*. *pneumophila* infection. RavK is able to cleave purified actin in reactions free of other proteins, indicating that the cleavage of actin by RavK does not require additional proteins from the host or *L*. *pneumophila* (**Fig 4D**). Interestingly, RavK exhibits a preference for non-muscle (85% β-actin and 15% γ-actin) over muscle actin (α-actin). The primary sequences of the three actin isoforms near the cleavage site are identical (**S6B Fig**), suggesting that the conformation of these actin isoforms at the cleavage site may vary, causing differences in the accessibility for the enzyme and differences in the cleavage efficiency. These results are in line with the fact that natural protozoan hosts of *L*. *pneumophila* such as *D*. *discoideum*, contain actin which shares a higher level identity of amino acid composition with human β-actin (93%) and γ-actin (93%) than with α-actin (89%).

Actin is a 375-amino acid polypeptide, which folds into two major domains. The two domains are separated by a deep cleft, in which relatively few interactions occur between the two domains. The polypeptide crosses the cleft twice in the middle of the cleft, dividing the cleft into two parts—upper and lower. The upper cleft is responsible for nucleotide binding, whereas the lower cleft is important for the interaction between actin subunits within the actin filaments. The lower cleft is lined by 11 predominantly hydrophobic residues including Leu349, Thr351[30]. Of note is that RavK cleaves actin at a site between Thr351 and Phec352, which locates on the outside end of the lower cleft, suggesting that cleavage of actin by RavK may interfere with the interaction between actin subunits in the filament and therefore inhibits the G-actin/F-actin transition. In agreement with this notion, in both the sedimentation and the pyrene-labeled actin nucleation assay, cleaved actin is defective in forming actin filaments (**Fig 8A–8D**).

During *L*. *pneumophila* infection, translocated RavK cleaves Flag-tagged actin into a smaller form (**Fig 5A**), the size difference between the full-length and cleaved actin is similar to that observed in *in vitro* cleavage assay (**Figs 4D** and **5A**), indicating that RavK likely cleaves actin in the same way under these conditions. Even though we can observe a clear cleaved Flag-tagged actin during the infection of *ΔravK* strain overexpressing RavK, we were unable to detect a reduction in endogenous actin during infection (**Fig 5D–5E**). Considering that only a small proportion of Flag-tagged actin is cleaved during infection (**Fig 5A**), and the fact that actin is very abundant in the cell, it is possible that the reduction of endogenous actin is too minute to be detected by the immunoblotting-based method. It is also possible that the host cell has a compensatory mechanism that once actin cytoskeleton is impaired due to a reduction of actin level, more actin will be synthesized to maintain the integrity of the actin cytoskeleton.

At least three *Legionella* Dot/Icm substrates have been shown to modulate the activity of components of the actin cytoskeleton. VipA is an actin nucleator, which localizes to actin rich regions and endosomes and interferes with the Multivesicular Body (MVB) pathway [13]; Ceg14 is a cytosolic protein, which inhibits actin polymerization by an unknown mechanism [12]; whereas LegK2 is a kinase, which localizes to the LCV and phosphorylates two subunits of the Arp2/3 complex to inhibit actin polymerization on the LCV [11]. Our demonstration of RavK as an effector that targets the actin cytoskeleton by cleaving actin indicates that *L*. *pneumophila* modulates this important host cellular component by diverse mechanisms. Given the essential role of actin in cellular processes, it is tempting to speculate that RavK targets to specific organelles such as the LCV, where it locally affects the function of actin in concert with effectors such as LegK2 to promote the biogenesis of the LCV. Unfortunately, we were unable to examine this hypothesis by directly staining for RavK during *L*. *pneumophila* infection due to low abundance of translocated RavK. Alternatively, translocated RavK may be quickly targeted for degradation by host proteases. Nevertheless, the low abundance of translocated RavK is consistent with its activity against an essential host protein. The distinct effects of multiple effectors on the actin cytoskeleton suggest the necessity of a coordinated modulation of the actin cytoskeleton at different levels. Whether these effectors directly balance the effects conferred by one or the other remains to be determined. Alternatively, given their different localization within the cell, it is plausible that these effectors regulate the actin cytoskeleton in an organelle-specific manner during *L*. *pneumophila* infection.

The fact that the *ravK/ceg14/legK2* triple mutant (Δ3) only has a relatively small growth defect in *D*. *discoideum* suggests the presence of additional Dot/Icm substrates targeting actin cytoskeleton, reiterating a significant functional redundancy among Dot/Icm substrates [45]. The lack of a strong defect of the Δ3 mutant in intracellular replication or in the cell biological events associated with *L*. *pneumophila* infection [14,15] makes it difficult to determine the benefit of targeting the actin cytoskeleton by the pathogen under current experimental conditions. Further studies are warranted to identify additional effectors that target the actin cytoskeleton and to elucidate their mechanisms of action.

## Materials and Methods

### Ethics statement

All animal use procedures were in strict accordance with the NIH Guide for the Care and Use of Laboratory Animals and were approved by the Purdue Animal Care and Use Committee (PACUC) (Protocol number 04–081).

### Bacterial, yeast strains and plasmid construction

Bacteria strains used in this study were listed in S1 Table. *E*. *coli* strains were grown in Luria broth (LB) medium and was supplemented with antibiotics when necessary. The *L*. *pneumophila* strains used in this study were derivatives of the Philadelphia-1 strain Lp02 [46]. *L*. *pneumophila* was grown and maintained in CYE medium according to a standard procedure [47]. The *ravK*, *ceg14* and *legK2* in-frame deletion mutants were constructed as described [48]. Briefly, for the construction of each knock-out plasmid, two pairs of primers were designed so that the target gene was replaced by 32 amino acids including the first and last 15 residues encoded by the gene and 2 residues encoded by the recognition site of *Bam*HI. For complementation experiments, the gene was expressed on the RSF1010-derived plasmid pZL507 [48]. For expression in mammalian cells, *ravK* or each of these genes was inserted into pEGFP-C1 (Clontech) or pFlag-CMV (Sigma). All the plasmids used in this study are listed in S2 Table and the sequences of all primers are in S3 Table.

### Yeast manipulation

Yeast strains used in this study were W303 [49] and its derivatives (S1 Table). Yeast strains were grown in yeast extract, peptone, dextrose medium (YPD) medium or appropriate amino acid dropout minimal media at 30°C [26]. Yeast transformation was performed with the lithium acetate method [50]. Yeast cell lysates for protein analysis were prepared as described [48].

### Yeast growth arrest assay

The ORF of *ravK* or its derivatives were inserted to pSB157 [51] to generate pGal::*ravK* (or pGal::*ravK* derivatives), which were digested with *Stu*I and transformed into yeast strain W303. To determine the yeast growth arrest induced by RavK, overnight cultures of relevant yeast strains grown in liquid selective medium containing glucose were serially diluted 5-fold, and 8μL of each dilution was spotted onto solid medium containing galactose or glucose. Plates were incubated at 30°C for 48 h before images were acquired.

### Tissue culture and transfection

COS-1, HEK293 and HEK293T cells were obtained from the American Type Culture Collection (Rockville, MD) and were cultured in Dulbecco’s modified minimum Eagle’s medium (DMEM) supplemented with 10% FBS fetal bovine/calf serum (FBS). Bone marrow-derived macrophages were prepared from A/J mice following the standard protocol [52]. For transient expression of exogenous proteins in HEK293T cells, 5 μL Lipofectamine 2000 (Invitrogen) was used to introduce 2.5 μg plasmids into mammalian cells per 6-well plate well at a cell confluency of 80%. For transient expression of exogenous proteins in COS-1 cells and HEK293 cells, 5 μL Lipofectamine 3000 and 5 μL P3000 (Invitrogen) were used to introduce 2.5 μg plasmids into cells per 6-well plate well at a cell confluency of 80%.

### Protein expression and purification

To purify His_6_-RavKΔC50 and His_6_-RavK_H95A_ΔC50, the appropriate gene fragments were inserted into the pQE30 plasmid (Qiagen) to generate pZL1221 and pZL1222, respectively. For protein production, 20-mL overnight culture of *E*. *coli* XL1blue harboring pZL1221 or pZL1222 were diluted into 800 mL LB medium (100 μg/mL ampicillin) and were allowed to grow at 37°C to OD_600_ = 0.6–0.8. After the IPTG was added to a concentration of 0.1 mM, the cultures were induced at 18°C for 16–18 h for protein expression. Bacterial cells were collected by centrifugation at 6,000*g* for 5 min and were lysed by sonication in the presence of protease inhibitors and 0.2% (wt/vol) TritonX-100. The soluble fractions were collected by centrifugation at 12,000*g* for 20 min and were incubated with Ni-NTA beads at 4°C for 2 h. Proteins bound to Ni^2+^-NTA beads were washed with 20 mM imidazole and were eluted with 300 mM imidazole. To remove imidazole, eluted proteins were dialyzed twice in 50 mM Tris·HCl (pH 8.0), 50 mM NaCl, 5% (vol/vol) glycerol and 1 mM DTT.

### Antibodies and immunoblotting

Polyclonal antibodies against RavK was generated at the Pocono Rabbit Farm and Laboratory using recombinant His_6_-tagged RavKΔC50 purified from *E*. *coli* to immunize a rabbit. The RavK antibody was affinity purified following a standard protocol [53]. The α-actin antibody C4 was purchased from MP Biochemicals (0869100) and was used at 1:5,000. The α-ICDH, α-GFP, α-PGK, α-Flag, α-tubulin were used as described in an earlier study [48]. Signals from each individual protein were detected by fluorescence dye-conjugated antibodies on an Odyssey detection system (Li-Cor).

### *In vitro* cleavage assay

Rabbit skeletal muscle actin (>99% pure) (Cytoskeleton) or Human platelet non-muscle actin (>99% pure) (Cytoskeleton) were incubated with RavKΔC50 or RavK_H95A_ΔC50 in G-actin buffer (5 mM Tris-HCl 8.0, 0.2 mM CaCl_2_, 0.2 mM ATP, 0.5 mM DTT) at 22°C for indicated time periods. Protein mixtures were analyzed by SDS-PAGE followed by Coomassie brilliant blue staining.

### Determination of the metal ion requirements of RavK

To obtain metal ion-free RavKΔ50, the protein was treated by 1 mM metal ion chelator 1,10-phenanthroline at 25°C for 20 min. After treatment, each of the seven different metal ions was added into an *in vitro* actin cleavage reaction containing 5 μg actin and 0.5 μg metal ion-free RavKΔC50 at a final concentration of 0.1 mM. *In vitro* reactions were allowed to proceed for 2 h before analysis by SDS-PAGE and Coomassie brilliant blue staining.

### Determination of the cleavage site by mass spectrometry

Actin that has been incubated with RavKΔC50 was separated by SDS-PAGE and the bands corresponding to the full-length actin and its cleavage products were excised and digested with trypsin. Peptides were analyzed in an Ekspert nanoLC system 400 (Eksigent) coupled to a 5600 TripleTOF mass spectrometer (AB Sciex). Peptides were separated in a capillary C18 column (75 μm x 15 cm, ChromXP C18-CL, 3 μm, 120 Å) with the following gradient: 1 min in 5% solvent B (Solvent A: 0.1% FA and solvent B: 80% ACN/ 0.1% FA), 5–35% solvent B in 60 min, 35–80% solvent B in 1 min, 6 min in 80% solvent B, 80–5% B in 1 min, and hold in 5% for 11 min. The flow rate was set at 200 nL/min and eluting peptides were directly analyzed in the mass spectrometer. Full-MS spectra were collected in the range of 400 to 2000 m/z and the top 50 most intense parent ions were submitted to fragmentation for 50 milliseconds using rolling-collision energy. Peptides with poor MS/MS spectra were targeted to data-independent acquisition, which enabled collecting high quality spectra. MS/MS spectra searched against the human SwissProt database (downloaded on July 09, 2013) using Paragon tool of Protein Pilot software (AB Sciex) considering biological post-translational modifications and matching peptides were inspected manually.

### Actin sedimentation and polymerization assay

30-μg G-actin was treated with 3-μg RavKΔC50, RavK_H95A_ΔC50 or left untreated in G-actin buffer at RT for 2 h. The obtained actin mixtures were precleared by centrifugation at 100,000*g* for 30 min and were used in actin co-sedimentation assays following an established protocol [34]. Briefly, the polymerization was initiated by adding 10× actin polymerization buffer (500 mM KCl, 10 mM MgCl_2_, 10 mM EGTA, 10 mM ATP) and was allowed to proceed for 60 min. The samples were subjected to ultracentrifugation at 100,000*g* for 40 min. Supernatants and pellets were analyzed by SDS-PAGE, followed by Coomassie brilliant blue staining. Pyrene-actin polymerization assay was performed as described by Schafer et al with minor modifications [54]. 60-μg non-muscle actin was treated by either His_6_-RavKΔC50, His_6_-RavK_H95A_ΔC50 or left untreated at RT for 2 h. His_6_-RavKΔC50, His_6_-RavK_H95A_ΔC50 were further removed from the cleaved products by passing through a Ni^2+^-NTA column. The flow-through was collected and precleared by centrifugation at 100,000*g* for 30 min. 2.7 μM precleared differentially-treated actin and 0.3 μM pyrene-labeled actin were mixed. Upon the addition of 10× polymerization buffer (500 mM KCl, 10 mM MgCl_2_, 10 mM EGTA, 100 mM imidazole HCl, pH 7.0), actin polymerization was monitored by measuring pyrene fluorescence intensity at 1s interval, using a PTI Alphascan spectrofluorimeter (Photon Technology International, South Brunswick, NJ). The excitation and emission wavelengths were set at 365 nm and 407 nm, respectively. Data were collected and processed in Excel (Microsoft) and the graph was made with Kaleidograph.

### Immunoprecipitation and *in vitro* cleavage

16–18 h after transfection, HEK293T or COS-1 cells were collected and lysed as previously described [48]. Approximately 1mg protein (in approximately 1 mL) was used for immunoprecipitation by adding 20 μL agarose beads coated with anti-Flag M2 antibody (Sigma). After incubating at 4°C for 3 h on a rotary shaker, the beads were washed with cold TBS for 4 times and proteins bound to the beads were eluted with 3×Flag peptides following manufacturer’s instructions. The eluted proteins were treated with 1 μg RavKΔC50 or RavK_H95A_ΔC50 in 50 μL G-actin buffer at 22°C for 2 h. The protein mixtures were resolved with SDS-PAGE gel, and detected by the M2 antibody. Immunoprecipitation with yeast lysates was carried out as described [48].

### Screening of *L*. *pneumophila* proteins that alter actin cytoskeleton

5×10^4^ COS-1 cells were seeded on 24-well plates and were allowed to grow at 37°C overnight. The next day, plasmids carrying full-length hypothetical *L*. *pneumophila* genes were introduced into COS-1 cells by Lipofectamine3000. 24 h later, cells were fixed in 4% paraformaldehyde in PBS at 25°C for 15 min, permeabilized with 0.2% Triton X-100 for 5 min, stained with Texas-red-conjugated phalloidin (1:500) at 25°C for 30 min, and subjected to imaging analysis. *L*. *pneumophila* genes that significantly altered the F-actin patterns of COS-1 cells were subjected to further analysis.

### F-actin and the average spread cell area quantitation

Fixed cells stained with Texas-red-conjugated phalloidin were subjected to imaging analysis under an Olympus X-81 fluorescence microscope. Images were acquired from a CoolSNAP HQ2 14-bit CCD camera (Photometrics) with identical parameters, and were similarly processed using the IPlab (BD Biosciences) and CellSens (Olympus Life Science) software package. We quantified the relative F-actin level following an established protocol [55]. Briefly, processed images were imported into ImageJ, and background was subtracted from each image. We then carefully outlined each cell by hand, and measured the integrated pixel density of each cell, which generated the average F-actin content per cell. We also measured the area occupied by each cell, which was shown as the average spread cell area.

### Intracellular bacterial growth assays

For infection experiments, *L*. *pneumophila* strains were grown to the post-exponential phase as measured by optical density of the culture (OD_600_ = 3.3–3.8) and judged by an increase in bacterial motility. For *L*. *pneumophila* intracellular growth assay, 4×10^5^ bone marrow-derived mouse macrophages or 5×10^5^ *D*. *discoideum* were seeded on 24-well plates and were infected with relevant *L*. *pneumophila* strains at an MOI = 0.05 at 37°C (for macrophage) or MOI = 0.1 at 25°C (for *D*. *discoideum*). At the indicated time points, cells were treated with 0.02% saponin for half an hour and the bacteria number was determined by enumerating colony-forming unit (CFU) of appropriately diluted saponin-soluble fractions.

### Determination of the cleavage of actin during infection

HEK293 cells were transfected to express 4×Flag-Actin and FCγRII with pJC119R [56,57] for 24 h with Lipofectamine 3000 (Life Technology). Bacteria of relevant *L*. *pneumophila* strains were opsonized with rabbit anti-*Legionella* antibodies [48] at 1:500 for 30 min before infecting the cells at an MOI of 50 for 2 h. Cleared lysates prepared from infected cells were subjected to immunoblotting with M2 antibody (Sigma). For experiments to determine the endogenous actin level during infection, HEK293 cells were transfected to express FCγRII for 24 h with Lipofectamine 3000 (Life Technology), bacterial infections were performed as described above. Cleared lysates prepared from infected cells were probed by immunoblotting with an actin-specific antibody.

### Determination of RavK translocation by *L*. *pneumophila*

HEK293 cells were transfected to express FCγRII for 24 h with Lipofectamine 3000 (Life Technology). Bacteria of relevant *L*. *pneumophila* strains were opsonized with rabbit anti-*Legionella* antibodies [48] at 1:500 for 30 min before infecting the cells at an MOI of 50 for 2 h. Infected cells were lysed with 0.02% saponin, which lyses membranes of mammalian cells but not of bacterial cells. The lysates were probed for RavK with a specific antibody. Translocation of the effector SidC [58] was probed as a control.

### Data quantitation and statistical analyses

Immunoblots were scanned with the Odyssey 3.0 (LI-COR Biosciences) and quantified with ImageJ. Statistical significance for all relevant data was calculated using the unpaired two-tailed Student *t* tests, with a *p* value <0.05 being considered as significant difference.

## Supporting Information

S1 Table Bacterial and yeast strains used in this study. (DOC)

S2 Table Plasmids used in this study. (DOC)

S3 Table Primers used in this study. (DOC)

S1 Fig Identification of *L*. *pneumophila* effectors that alter the architecture of the actin cytoskeleton of mammalian cells. COS-1 cells were transfected by the indicated plasmids for 24 hours and cells were fixed and subjected to staining with Texas-red-conjugated phalloidin. Representative images were shown. Bar, 20 μm.
(TIFF)

S2 Fig The H_95_E_XX_H_99_ motif is essential for toxicity of RavK toward mammalian cells. A. HEK293T cells were transfected to express GFP fusion of RavK, RavK_H95A,_ RavK_E96A_ or RavK_H99A_ for 16 h and the images were acquired by a fluorescence microscope. Bar, 50 μm. **B.** Expression of GFP fusions in samples from A. Total cell lysates resolved by SDS-PAGE were probed with antibodies specific for GFP (for the GFP fusion to RavK and its derivatives) and for tubulin as a loading control.
(TIFF)

S3 Fig RavK reduces the actin level in HEK293T cells. **A.** Expression of RavK reduces the level of actin in HEK293T cells. Cell transfection and immunoblotting were performed similarly as Fig 4A. **B.** Quantification of the band intensity ratio of actin versus tubulin as described in Fig 4B. All results are from three independent experiments. Error bars represent SEM. **, *p*<0.01, ***, *p*<0.001. **C.** Recombinant RavKΔC50 cleaves actin in HEK293 cell lysates. Cleavage and immunoblotting were performed as described for Fig 4C.
(TIFF)

S4 Fig Deletion of 50 residues from the C-terminal end of RavK allowed the purification of active protein. **A**. Expression of RavK and RavKΔC50 in *E*. *coli*. Note that RavKΔC50 is more soluble than RavK. T, Total lysate; P, Pellet; S, Supernatant; E, Elution. **B.** RavKΔC50 but not RavKΔC100 inhibits yeast growth. Yeast toxicity assay was performed as described in Fig 3B. **C.** Expression of RavK and indicated mutants in yeast. Total proteins of the indicated yeast strains induced with galactose as described in Fig 3C were probed by immunoblotting for RavK and the PGK kinase was probed as a loading control. **D-E.** The toxicity of RavKΔC50 to mammalian cells. GFP fusion of full-length or RavKΔC50 was expressed in 293T cells and the images were acquired 16 h after transfection (D), the expression of the fusions were probed with an antibody specific for GFP (E) and tubulin was probed as a loading control.
(TIFF)

S5 Fig The effects of several divalent metal ions on the activity of metal-free RavK. The indicated metal ions were individually added to reactions containing actin and His_6_-RavKΔC50 treated with 1,10-phenanthroline. 2 h after incubation, the enzymatic activity was assessed by detecting the production of cleaved actin after SDS-PAGE and Coomassie brilliant blue staining. Similar results were obtained in three independent experiments.
(TIFF)

S6 Fig Preparation of samples for mass spectrometry analysis and the sequence alignment of the RavK recognition site from different forms of actin. **A.** Non-muscle actin was incubated with RavK for 1 h, and the protein mixtures were resolved by SDS-PAGE, followed by Coomassie brilliant blue staining. Both upper and lower bands were excised and analyzed by mass spectrometry. **B.** Sequence alignment of yeast actin and the three human actin isoforms. Red box highlighted the six residues examined in Fig 6C.
(TIFF)

S7 Fig Deletion of *ravK* did not affect intracellular growth of *L*. *pneumophila*. A mutant lacking *ravK* or a mutant lacking both *ravK* and *ceg14* did not show any defects in intracellular growth. **A.** Intracellular growth in primary bone marrow-derived macrophages from A/J mice. **B.** Intracellular growth in *D*. *discoideum*. **C.** A mutant lacking *ravK*, *ceg14* and *legK2* did not show a more severe defect in intracellular growth than the *legK2* deletion mutant in *D*. *discoideum*. **D.** The growth defect of the triple mutant lacking *ravK*, *ceg14* and *legK2* can be complemented by *legK2* but not by *ceg14* or *ravK*. In each case, the host cells were challenged with the indicated bacterial strains grown to post-exponential phase and the total bacterial counts at the indicated time points were determined by plating appropriate dilutions of lysates onto bacteriological media to determine the CFU. Results shown are from one representative experiment done in triplicate. Similar results were obtained in three independent experiments.
(TIFF)
