# Supplementary material for: A Legionella Effector Disrupts Host Cytoskeletal Structure by Cleaving Actin
Source: PLoS Pathog. 2017 Jan 27;13(1):e1006186. doi: 10.1371/journal.ppat.1006186 (PMC5298343; doi:10.1371/journal.ppat.1006186)
Supplement: S1 Table — (DOC) [file ppat.1006186.s001.doc]

| S1 Table Bacterial and yeast strains used in this study | |  |
| --- | --- | --- |
| Strains | Genotype, relevant markers | Reference |
| *E. coli* |  | Stratagene |
| XL1-Blue | *recA1* endA1 *gyrA*96 *thi-1* *hsd*R17 *sup*E44 *relA*1 *lac* [*F*' *proAB lacI*^q^*ZM15* Tn*10*(Tet^r^)] |
| DH5α(λpir) | supE44 d*lacU169*(φ80*lacZ*Δ*M15*) *hsdR17 recA1 endA1 gyrA96 thi-1 relA1 pir tet*::*Mu recA* | Our collection |
| *L. pneumophila* |  |  |
| Lp02 | Philadelphia-1 *rpsL hsdR thyA* |  |
| Lp03 | Lp02(*dotA*^-^) |  |
| Lp02(pJB908) | Lp02(pJB908) |  |
| Lp03(pJB908) | Lp03(pJB908) |  |
| ZL1101 | Lp02*∆ravK* | This study |
| ZL1102 | Lp02*∆ravK* (pJB908) | This study |
| ZL1103 | Lp02*∆ravK* (pZL507::*ravK*) | This study |
| ZL1104 | Lp02*∆ravK∆ceg14* | This study |
| ZL1105 | Lp02*∆ravK∆ceg14* (pJB908) | This study |
| ZL1106 | Lp02*∆ravK∆ceg14∆legK2* | This study |
| ZL1107 | Lp02*∆ravK∆ceg14∆legK2* (pJB908) | This study |
| ZL1108 | Lp02*∆ravK∆ceg14∆legK2* (pZL507::*ravK*) | This study |
| ZL1109 | Lp02*∆ravK∆ceg14∆legK2* (pZL507::*ceg14*) | This study |
| ZL1110 | Lp02*∆ravK∆ceg14∆legK2* (pZL507::*legK2*) | This study |
|  |  |  |
| Yeast |  |  |
| W303 | *MATa/MATα {leu2-3,112 trp1-1 can1-100 ura3-1 ade2-1 his3-11,15} [phi+]* |  |

**Reference:**

1. Berger KH, Isberg RR (1993) Two distinct defects in intracellular growth complemented by a single genetic locus in Legionella pneumophila. Mol Microbiol 7: 7-19.

2. Liu Y, Luo ZQ (2007) The Legionella pneumophila effector SidJ is required for efficient recruitment of endoplasmic reticulum proteins to the bacterial phagosome. Infect Immun 75: 592-603.

3. Fan HY, Cheng KK, Klein HL (1996) Mutations in the RNA polymerase II transcription machinery suppress the hyperrecombination mutant hpr1 delta of Saccharomyces cerevisiae. Genetics 142: 749-759.
