# Supplementary material for: A Legionella Effector Disrupts Host Cytoskeletal Structure by Cleaving Actin
Source: PLoS Pathog. 2017 Jan 27;13(1):e1006186. doi: 10.1371/journal.ppat.1006186 (PMC5298343; doi:10.1371/journal.ppat.1006186)
Supplement: S2 Table — (DOC) [file ppat.1006186.s002.doc]

| S2 Table Plasmids used in this study | | |
| --- | --- | --- |
|
| Plasmid | Relevant phenotypes | Sources |
| pSR47s | *ori*R6K, *ori*T RP4, Kan^R^, *SacB* |  |
| pQE30 | Amp | Qiagen |
| pJB908 | Amp, *thy*^+^ |  |
| pZL507 | For expression His_6_-tagged protein *L. pneumophila* |  |
| pEGFPC-1 | For expressing C-terminal GFP fusion proteins | Clontech |
| pYES/NT A | Yeast expression vector with N-terminal tag | Invitrogen |
| pSB157m | Amp, *ura^+^,* GAL promoter |  |
| p415-ADH | Amp, *leu*^+^, ADH promoter |  |
| pZLΔ*ravK* | Construct used for in-frame deletion of *ravK* | This study |
| pZLΔ*ceg14* | Construct used for in-frame deletion of *ceg14* |  |
| pZLΔ*legK2* | Construct used for in-frame deletion of *legK2* | This study |
| pZL1201 | pYES/NT A::*ravK* | This study |
| pZL1202 | pYES/NT A::*ravK*(H95A) | This study |
| pZL1203 | pYES/NT A::*ravK*(E96A) | This study |
| pZL1204 | pYES/NT A::*ravK*(H99A) | This study |
| pZL1205 | pEGFPc1::lpg0483 | This study |
| pZL1206 | pEGFPc1::lpg0944 | This study |
| pZL1207 | pEGFPc1::lpg1290 | This study |
| pZL1208 | pEGFPc1::lpg1798 | This study |
| pZL1209 | pEGFPc1::lpg1961 | This study |
| pZL1210 | pEGFPc1::lpg2322 | This study |
| pZL1211 | pEGFPc1::lpg2603 | This study |
| pZL1212 | pEGFPc1::*ravK* | This study |
| pZL1213 | pEGFPc1::*ravK*(H95A) | This study |
| pZL1214 | pEGFPc1::*ravK*(E96A) | This study |
| pZL1215 | pEGFPc1::*ravK*(E96A) | This study |
| pZL1216 | pEGFPc1::*ravK*ΔC50 | This study |
| pZL1217 | pSB157m::*ravK* | This study |
| pZL1218 | pSB157m::*ravK*ΔC50 | This study |
| pZL1219 | pSB157m::*ravK*ΔC50(H95A) | This study |
| pZL1220 | pSB157m::*ravK*ΔC100 | This study |
| pZL1221 | pQE30::*ravK*ΔC50 | This study |
| pZL1222 | pQE30::*ravK*ΔC50(H95A) | This study |
| pZL1223 | pCMV-1XFlag-β-actin | This study |
| pZL1224 | pCMV-1XFlag-β-actin(L349A) | This study |
| pZL1225 | pCMV-1XFlag-β-actin(S350A) | This study |
| pZL1226 | pCMV-1XFlag-β-actin(T351A) | This study |
| pZL1227 | pCMV-1XFlag-β-actin(F352A) | This study |
| pZL1228 | pCMV-1XFlag-β-actin(Q353A) | This study |
| pZL1229 | pCMV-1XFlag-β-actin(Q354A) | This study |

**Reference:**

1. Dumenil G, Isberg RR (2001) The Legionella pneumophila IcmR protein exhibits chaperone activity for IcmQ by preventing its participation in high-molecular-weight complexes. Mol Microbiol 40: 1113-1127.

2. Bardill JP, Miller JL, Vogel JP (2005) IcmS-dependent translocation of SdeA into macrophages by the Legionella pneumophila type IV secretion system. Mol Microbiol 56: 90-103.

3. Xu L, Shen X, Bryan A, Banga S, Swanson MS, et al. (2010) Inhibition of host vacuolar H+-ATPase activity by a Legionella pneumophila effector. PLoS Pathog 6: e1000822.

4. Tan YH, Luo ZQ (2011) Legionella pneumophila SidD is a deAMPylase that modifies Rab1. Nature 475: 506-U102.

5. Mumberg D, Muller R, Funk M (1995) Yeast vectors for the controlled expression of heterologous proteins in different genetic backgrounds. Gene 156: 119-122.

6. Guo Z, Stephenson R, Qiu J, Zheng S, Luo ZQ (2014) A Legionella effector modulates host cytoskeletal structure by inhibiting actin polymerization. Microbes Infect 16: 225-236.
