# Supplementary material for: A Legionella Effector Disrupts Host Cytoskeletal Structure by Cleaving Actin
Source: PLoS Pathog. 2017 Jan 27;13(1):e1006186. doi: 10.1371/journal.ppat.1006186 (PMC5298343; doi:10.1371/journal.ppat.1006186)
Supplement: S3 Table — (DOC) [file ppat.1006186.s003.doc]

| S3 Table Primers used in this study | |  |
| --- | --- | --- |
|  | |  |
| Primer | Sequence (Restriction enzyme sites are underlined) | Note |
| PL1101 | CTGGGATCCATGGTAAGTTTGGAGCAT | ravK5’BamHI |
| PL1102 | CTGGTCGACTTATATATCAAGCTTTAT | ravK3’SalI |
| PL1103 | CTGAAGCTTATGGATGATGATATCGCCGC | β-actin5’HindIII |
| PL1104 | CTGCTCGAGCTAGAAGCATTTGCGGTGGA | β-actin3’XhoI |
| PL1105 | ATACTCGAGAAATGCCATGCTTAATAA | ravK up XhoI knock-out |
| PL1106 | ATAGGATCCCCTACATTCCGAAATAAG | ravK up BamHI knock-out |
| PL1107 | ATAGGATCCGAGCATTTTCATTCTCCT | ravK down BamHI knockout |
| PL1108 | ATAGCGGCCGCATAATGGAATTGTTTACT | ravK down NotI knock-out |
| PL1109 | CTGGTCGACTAATCGTTTATGATAGTAAA | legK2 up SalI knock-out |
| PL1110 | CTGGGATCCATCTTGATGTAAAGGTTGTT | legK2 up BamHI knock-out |
| PL1111 | CTGGGATCCAAGCAACCTATATTTACCCC | legK2 down BamHI knockout |
| PL1112 | CTGGAGCTCATTTTATCCTGCTGAATACC | legK2 down SacI knock-out |
| PL1113 | ttaaaagcatgaccagtttcagcaacaatagca  ccaataattttgatcgcc | ravK(H95A)-1 |
| PL1114 | ggcgatcaaaattattggtgctattgttgctgaa  actggtcatgcttttaa | ravK(H95A)-2 |
| PL1115 | ccacattaaaagcatgaccagttgcatgaacaa  tagcaccaataatt | ravK(E96A)-1 |
| PL1116 | aattattggtgctattgttcatgcaactggtcat  gcttttaatgtgg | ravK(E96A)-2 |
| PL1117 | cagccacattaaaagcagcaccagtttcatgaa  caatagcaccaa | ravK(H99A)-1 |
| PL1118 | ttggtgctattgttcatgaaactggtgctgctttt  aatgtggctg | ravK(H99A)-2 |
| PL1119 | gctggaaggtggacgacgaggccaggatgg | β-actin(L349S)-1 |
| PL1120 | ccatcctggcctcgtcgtccaccttccagc | β-actin(L349S)-1 |
| PL1121 | gctggaaggtggccagcgaggccag | β-actin(S350A)-1 |
| PL1122 | ctggcctcgctggccaccttccagc | β-actin(S350A)-1 |
| PL1123 | atctgctggaaggcggacagcgaggcc | β-actin(T351A)-1 |
| PL1124 | ggcctcgctgtccgccttccagcagat | β-actin(T351A)-1 |
| PL1125 | atccacatctgctgggcggtggacagcgaggc | β-actin(F352A)-1 |
| PL1126 | gcctcgctgtccaccgcccagcagatgtggat | β-actin(F352A)-2 |
| PL1127 | ctcgctgtccaccttcgcgcagatgtggatcagc | β-actin(Q353A)-1 |
| PL1128 | gctgatccacatctgcgcgaaggtggacagcgag | β-actin(Q353A)-1 |
| PL1129 | cttgctgatccacatcgcctggaaggtggacagc | β-actin(Q354A)-1 |
| PL1130 | gctgtccaccttccaggcgatgtggatcagcaag | β-actin(Q354A)-1 |
