# Supplementary figures and images for: A Legionella Effector Disrupts Host Cytoskeletal Structure by Cleaving Actin
Source: PLoS Pathog. 2017 Jan 27;13(1):e1006186. doi: 10.1371/journal.ppat.1006186 (PMC5298343; doi:10.1371/journal.ppat.1006186)

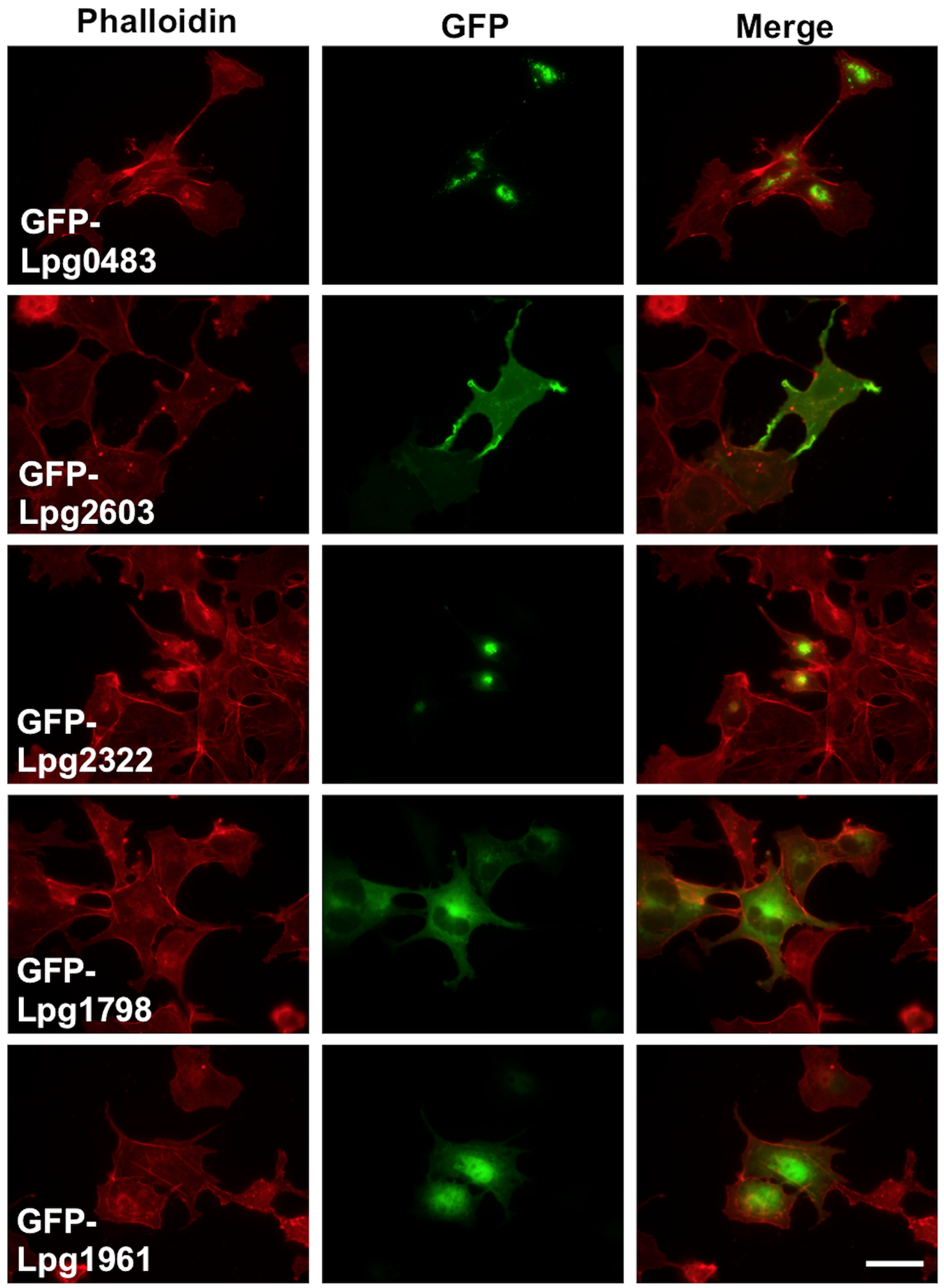

Supplement: S1 Fig — COS-1 cells were transfected by the indicated plasmids for 24 hours and cells were fixed and subjected to staining with Texas-red-conjugated phalloidin. Representative images were shown. Bar, 20 μm. (TIFF) [file ppat.1006186.s004.tiff]

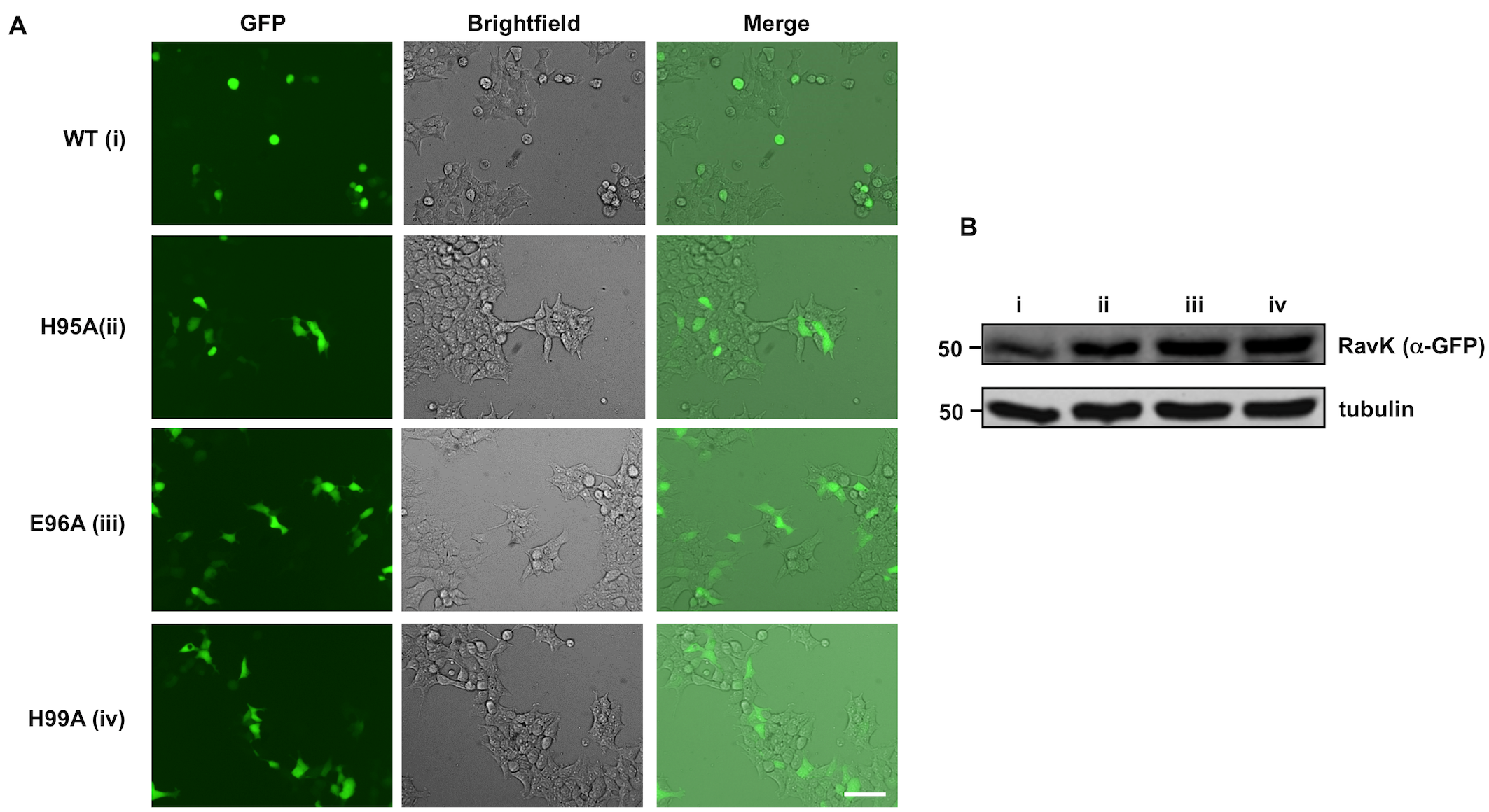

Supplement: S2 Fig — A. HEK293T cells were transfected to express GFP fusion of RavK, RavKH95A, RavKE96A or RavKH99A for 16 h and the images were acquired by a fluorescence microscope. Bar, 50 μm. B. Expression of GFP fusions in samples from A. Total cell lysates resolved by SDS-PAGE were probed with antibodies specific for GFP (for the GFP fusion to RavK and its derivatives) and for tubulin as a loading control. (TIFF) [file ppat.1006186.s005.tiff]

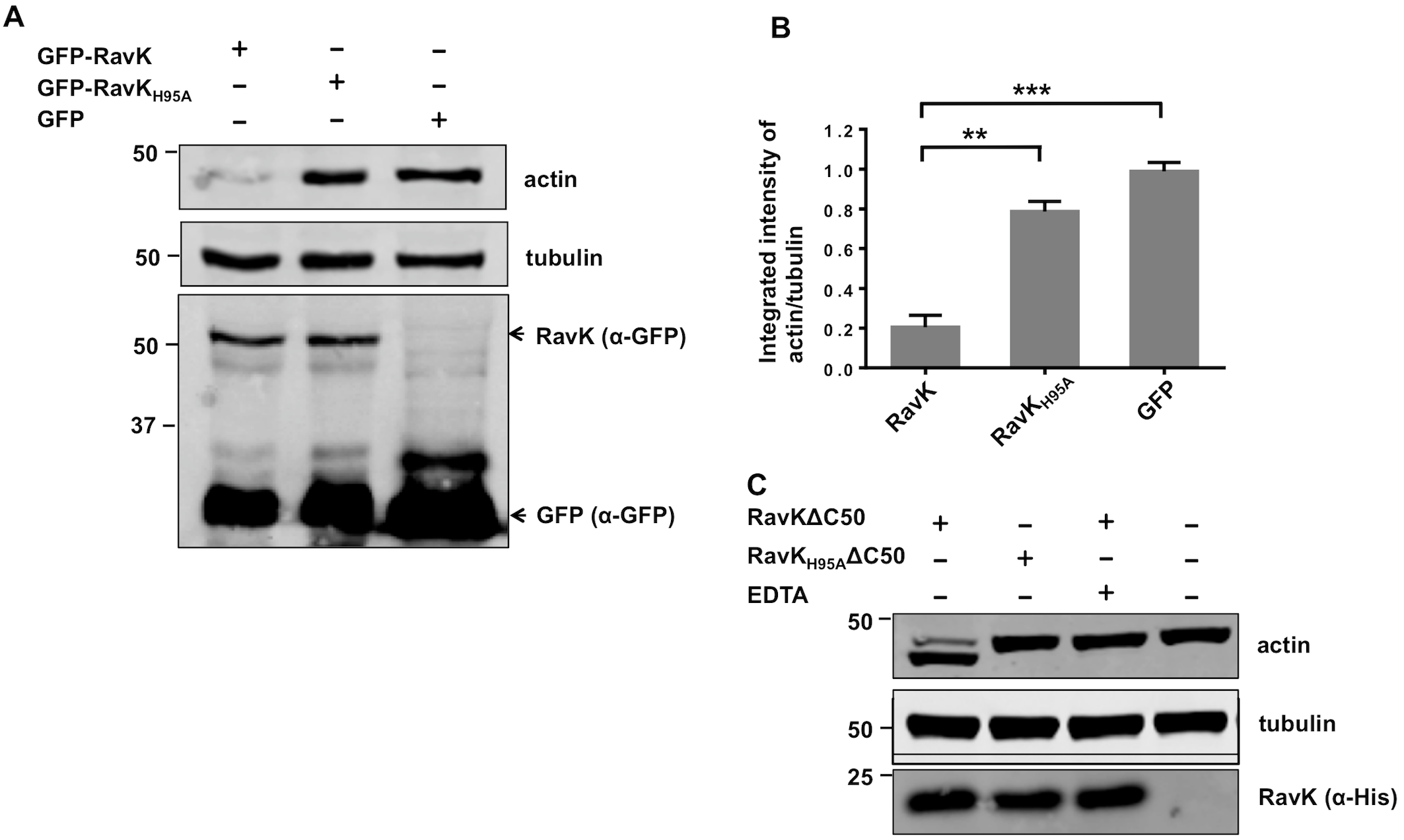

Supplement: S3 Fig — A. Expression of RavK reduces the level of actin in HEK293T cells. Cell transfection and immunoblotting were performed similarly as Fig 4A. B. Quantification of the band intensity ratio of actin versus tubulin as described in Fig 4B. All results are from three independent experiments. Error bars represent SEM. **, p<0.01, ***, p<0.001. C. Recombinant RavKΔC50 cleaves actin in HEK293 cell lysates. Cleavage and immunoblotting were performed as described for Fig 4C. (TIFF) [file ppat.1006186.s006.tiff]

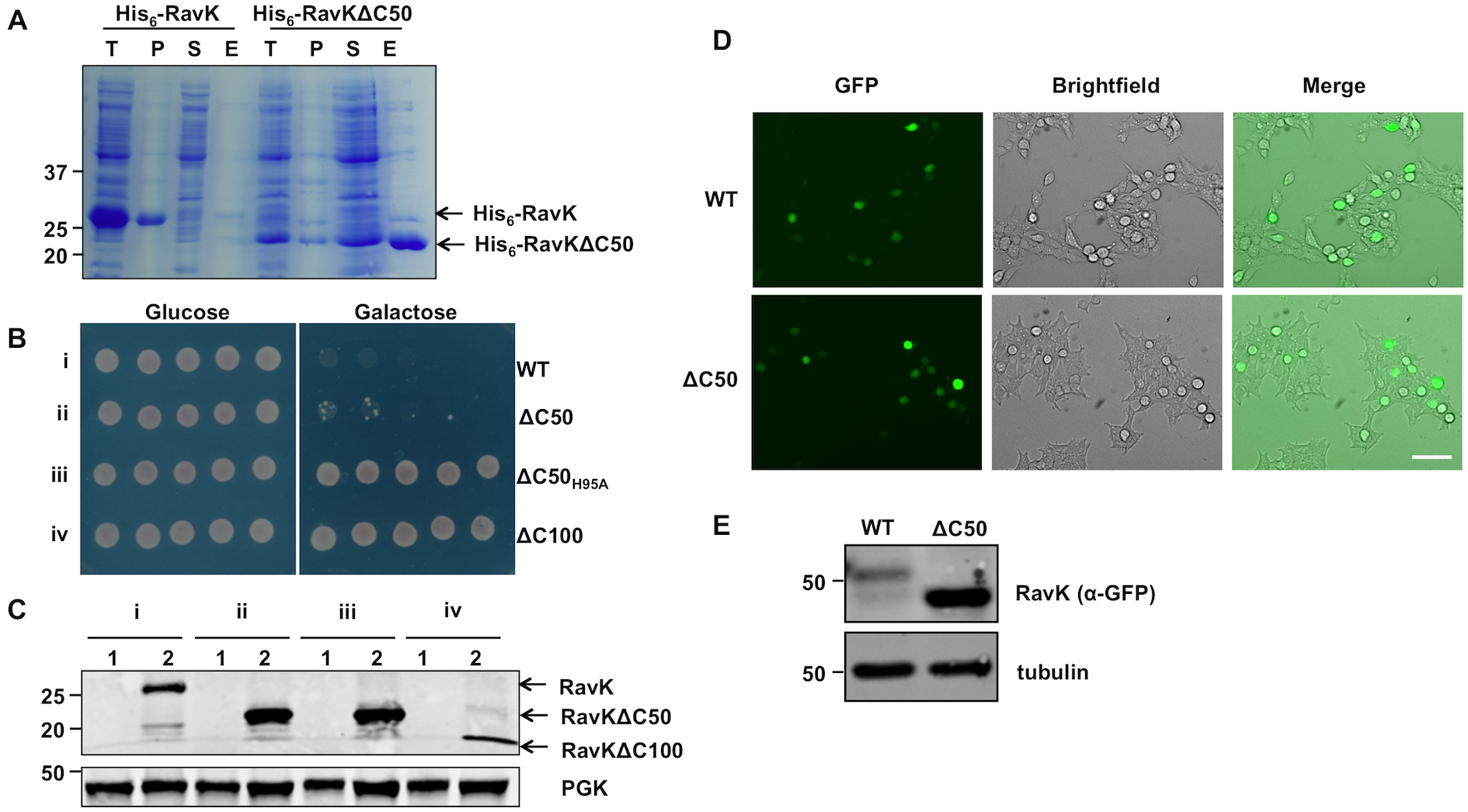

Supplement: S4 Fig — A. Expression of RavK and RavKΔC50 in E. coli. Note that RavKΔC50 is more soluble than RavK. T, Total lysate; P, Pellet; S, Supernatant; E, Elution. B. RavKΔC50 but not RavKΔC100 inhibits yeast growth. Yeast toxicity assay was performed as described in Fig 3B. C. Expression of RavK and indicated mutants in yeast. Total proteins of the indicated yeast strains induced with galactose as described in Fig 3C were probed by immunoblotting for RavK and the PGK kinase was probed as a loading control. D-E. The toxicity of RavKΔC50 to mammalian cells. GFP fusion of full-length or RavKΔC50 was expressed in 293T cells and the images were acquired 16 h after transfection (D), the expression of the fusions were probed with an antibody specific for GFP (E) and tubulin was probed as a loading control. (TIFF) [file ppat.1006186.s007.tiff]

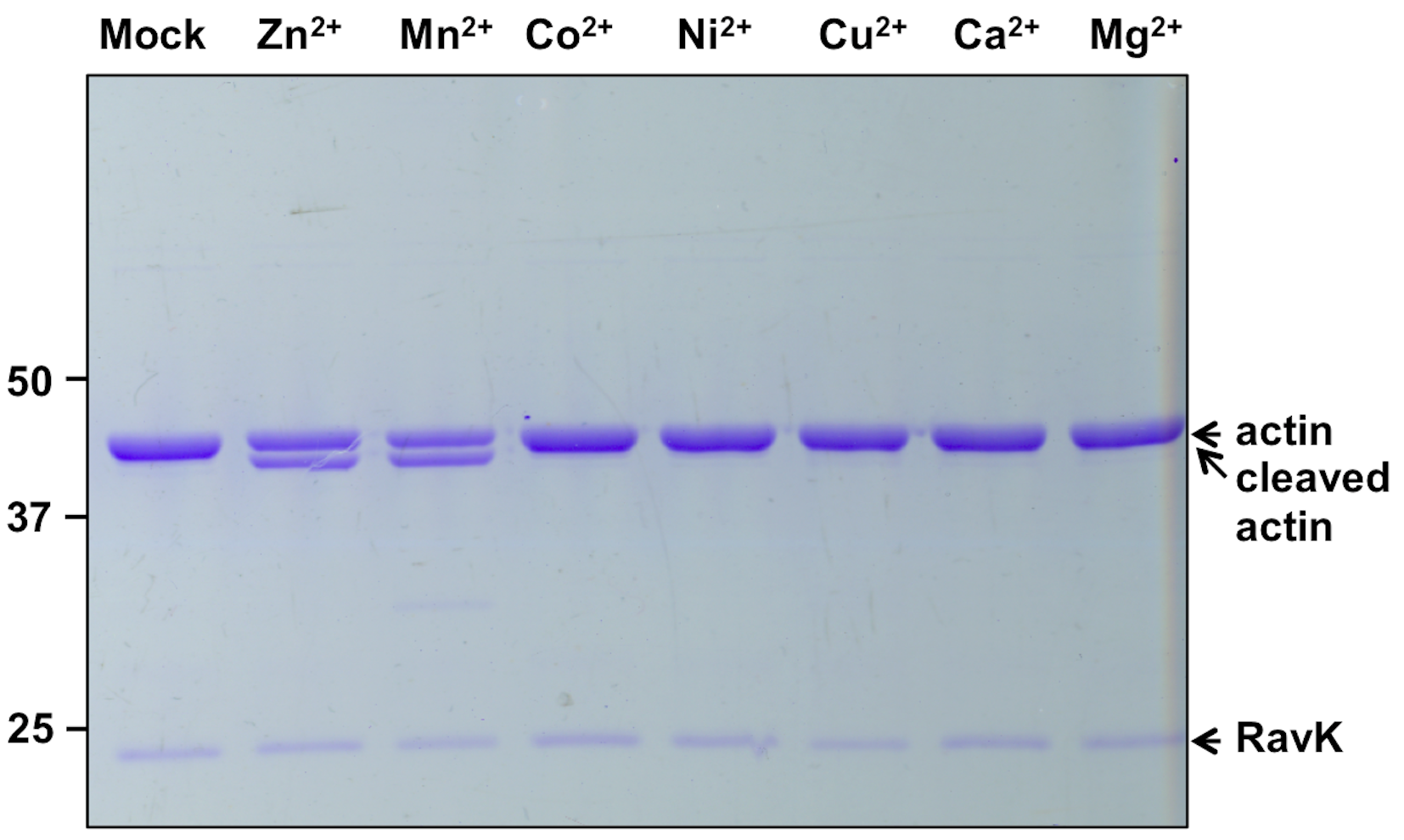

Supplement: S5 Fig — The indicated metal ions were individually added to reactions containing actin and His6-RavKΔC50 treated with 1,10-phenanthroline. 2 h after incubation, the enzymatic activity was assessed by detecting the production of cleaved actin after SDS-PAGE and Coomassie brilliant blue staining. Similar results were obtained in three independent experiments. (TIFF) [file ppat.1006186.s008.tiff]

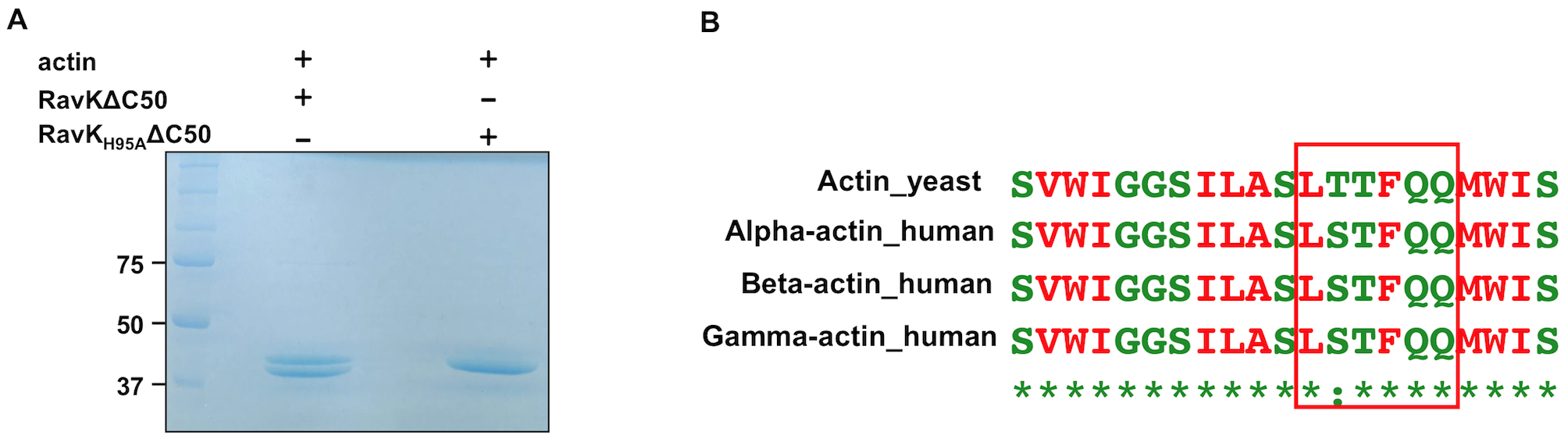

Supplement: S6 Fig — A. Non-muscle actin was incubated with RavK for 1 h, and the protein mixtures were resolved by SDS-PAGE, followed by Coomassie brilliant blue staining. Both upper and lower bands were excised and analyzed by mass spectrometry. B. Sequence alignment of yeast actin and the three human actin isoforms. Red box highlighted the six residues examined in Fig 6C. (TIFF) [file ppat.1006186.s009.tiff]

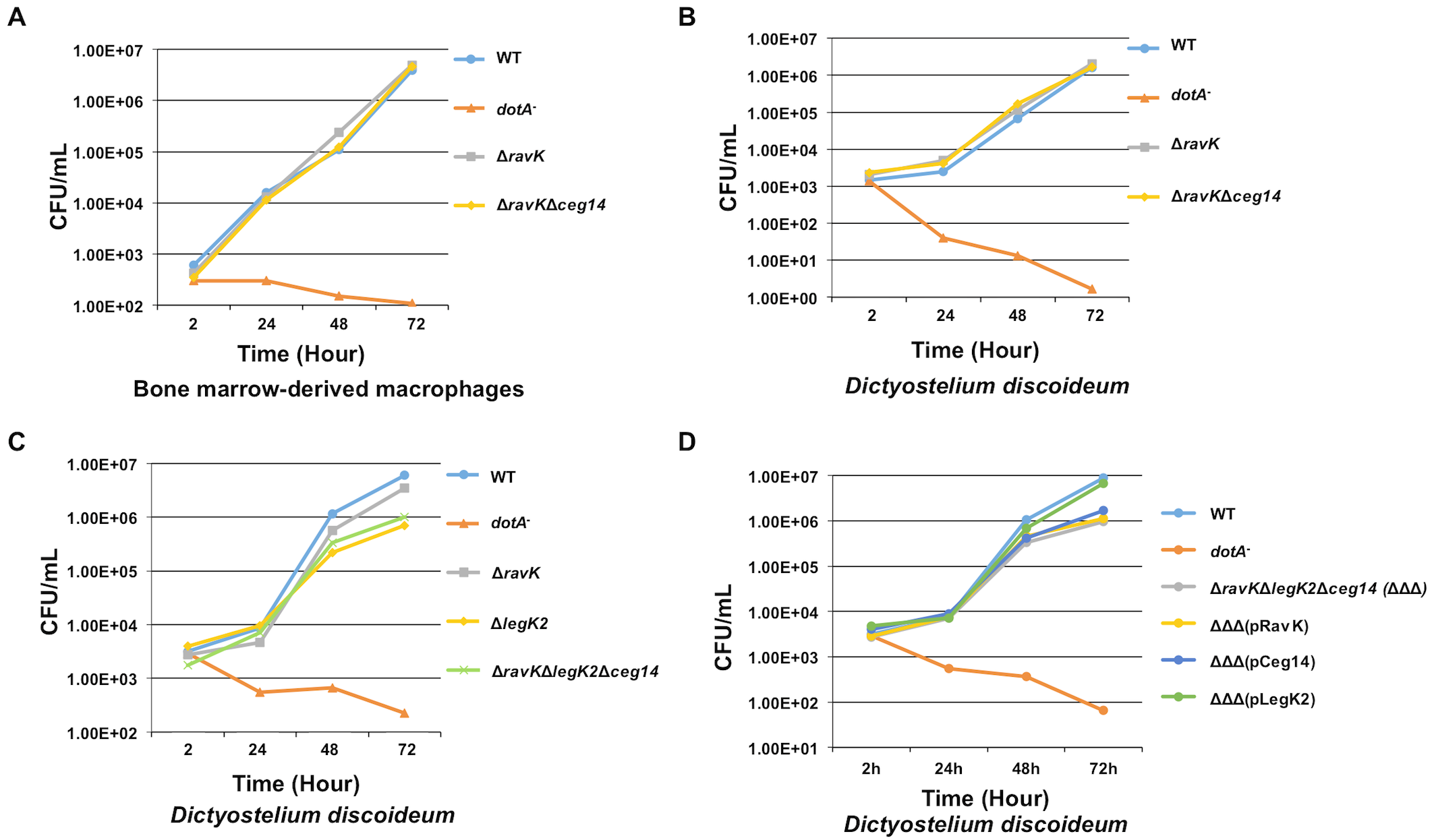

Supplement: S7 Fig — A mutant lacking ravK or a mutant lacking both ravK and ceg14 did not show any defects in intracellular growth. A. Intracellular growth in primary bone marrow-derived macrophages from A/J mice. B. Intracellular growth in D. discoideum. C. A mutant lacking ravK, ceg14 and legK2 did not show a more severe defect in intracellular growth than the legK2 deletion mutant in D. discoideum. D. The growth defect of the triple mutant lacking ravK, ceg14 and legK2 can be complemented by legK2 but not by ceg14 or ravK. In each case, the host cells were challenged with the indicated bacterial strains grown to post-exponential phase and the total bacterial counts at the indicated time points were determined by plating appropriate dilutions of lysates onto bacteriological media to determine the CFU. Results shown are from one representative experiment done in triplicate. Similar results were obtained in three independent experiments. (TIFF) [file ppat.1006186.s010.tiff]
